# Recent advances in the syntheses of anthracene derivatives

**DOI:** 10.3762/bjoc.17.131

**Published:** 2021-08-10

**Authors:** Giovanni S Baviera, Paulo M Donate

**Affiliations:** 1 Departamento de Química, Faculdade de Filosofia, Ciências e Letras de Ribeirão Preto, Universidade de São Paulo, 14040-091, Ribeirão Preto, SP, Brazil

**Keywords:** anthracenes, anthraquinones, Friedel–Crafts cyclization, intramolecular cyclization, metal-catalyzed

## Abstract

Anthracene and anthracene derivatives have been extensively studied over the years because of their interesting photophysical, photochemical, and biological properties. They are currently the subject of research in several areas, which investigate their use in the biological field and their application in OLEDs, OFETs, polymeric materials, solar cells, and many other organic materials. Their synthesis remains challenging, but some important preparative methods have been reported, especially in the last decade. This review presents an update of the recent strategies that have been employed to prepare anthracene derivatives. It encompasses papers published over the last twelve years (2008–2020) and focuses on direct and indirect methods to construct anthracene and anthraquinone frameworks.

## Introduction

**Figure 1:**
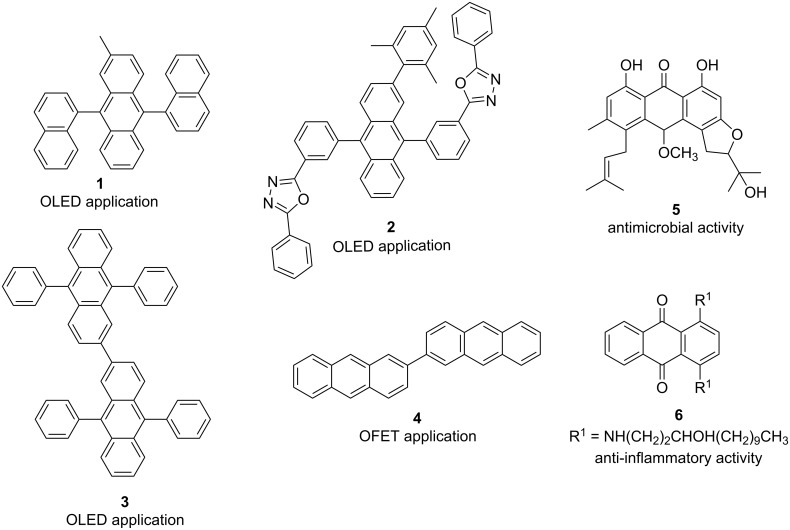
Examples of anthracene derivatives and their applications.

Anthracene is an important aromatic hydrocarbon consisting of three linearly fused benzene rings. Because of their extended aromatic and conjugated π-system, anthracene derivatives possess interesting photochemical and photophysical properties [1–3], as well as gelling ability [4]. These important properties make them relevant for the development and application of several organic materials, such as organic light-emitting diodes (OLEDs) [5], organic field-effect transistors (OFETs) [6], polymeric materials [7], and other kinds of materials [8–10]. For example, OLEDs fabricated with 9,10-diphenylanthracene derivatives **1** and **2** are blue light emitters [11–12], the 2,2’-bianthracene derivative **3** provides a green and fluorescent OLED [13], 2,2’-bianthracenyl (**4**) has been employed as an organic semiconductor in an OFET device [14], and di-*n*-alkoxyanthracenes have gelling properties with diverse solvents, mainly alkanes and alcohols [4]. Furthermore, anthracene derivatives display useful biological activities; for instance, the anthraquinone derivatives **5** and **6** exert antimicrobial and anti-inflammatory activity, respectively (Figure 1) [15–16].

Despite some difficulties and limitations, a number of synthetic methods for preparing anthracene derivatives has been reported over the years. The most familiar methods to obtain substituted anthracenes include Friedel–Crafts reactions [17], Elbs reaction [18], aromatic cyclodehydration [19–20], Bradsher-type reactions from diarylmethanes [21–23], and, more recently, metal-catalyzed reactions with alkynes [24–25]. Numerous synthetic routes have also been reported for the synthesis of anthraquinones [26–29]. On the other hand, preparative methods for dibenzo[*a*,*h*]anthracene derivatives are less common, mainly relying on the cyclization of the corresponding lactone derivative followed by sequential modifications [30], photocyclization of divinylterphenyl derivatives [31], tandem radical cyclization of (*Z,Z*)-1,4-bis(2-iodostyryl)benzene derivatives [32], and ring-closing olefin metathesis of tetravinylterphenyls [33] as the best-known synthetic routes.

Herein, we have classified the synthetic methods into three general categories: synthesis of substituted anthracene frameworks, synthesis of benzanthracene and dibenzanthracene derivatives, and synthesis of anthraquinone derivatives. We will focus on the construction of the anthracene and anthraquinone frameworks published in the last twelve years (2008–2020); methods for simple modifications of the anthracene and anthraquinone rings will be excluded. To the best of our knowledge, this is the first review involving the synthesis of anthracene derivatives spanning this period.

## Review

### Synthesis of substituted anthracene frameworks

#### Metal-catalyzed reactions with alkynes

**Scheme 1:**
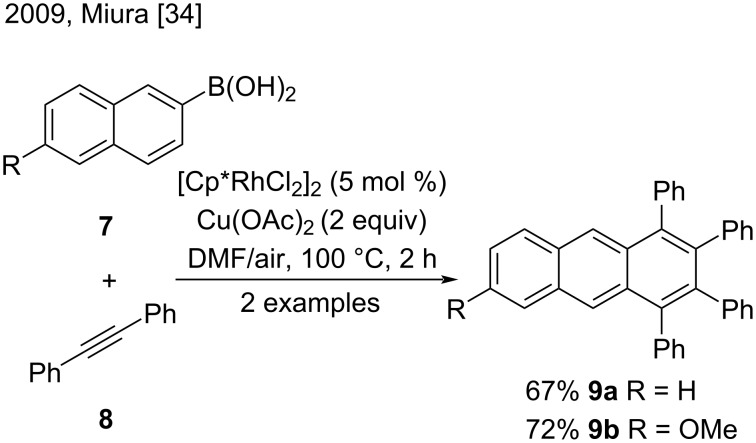
Rhodium-catalyzed oxidative coupling reactions of arylboronic acids with internal alkynes.

Metal-catalyzed reactions with alkynes have gained attention in the last years and have provided new methodologies to prepare anthracene derivatives. In 2009, Miura and co-workers were the first to obtain substituted anthracenes selectively by homologations with monofunctionalized naphthyl substrates. These authors demonstrated that the rhodium-catalyzed oxidative 1:2 coupling reactions of arylboronic acids **7** with alkyne **8** occurred in the presence of a copper–air oxidant, to give the corresponding 1,2,3,4-tetrasubtituted anthracene derivatives **9a** and **9b** (Scheme 1) [34]. Although the scope of the reaction was broader for 1,2,3,4-substituted naphthalenes, the authors developed a potentially applicable methodology to synthesize substituted anthracenes and other polysubstituted fused aromatic compounds.

**Scheme 2:**
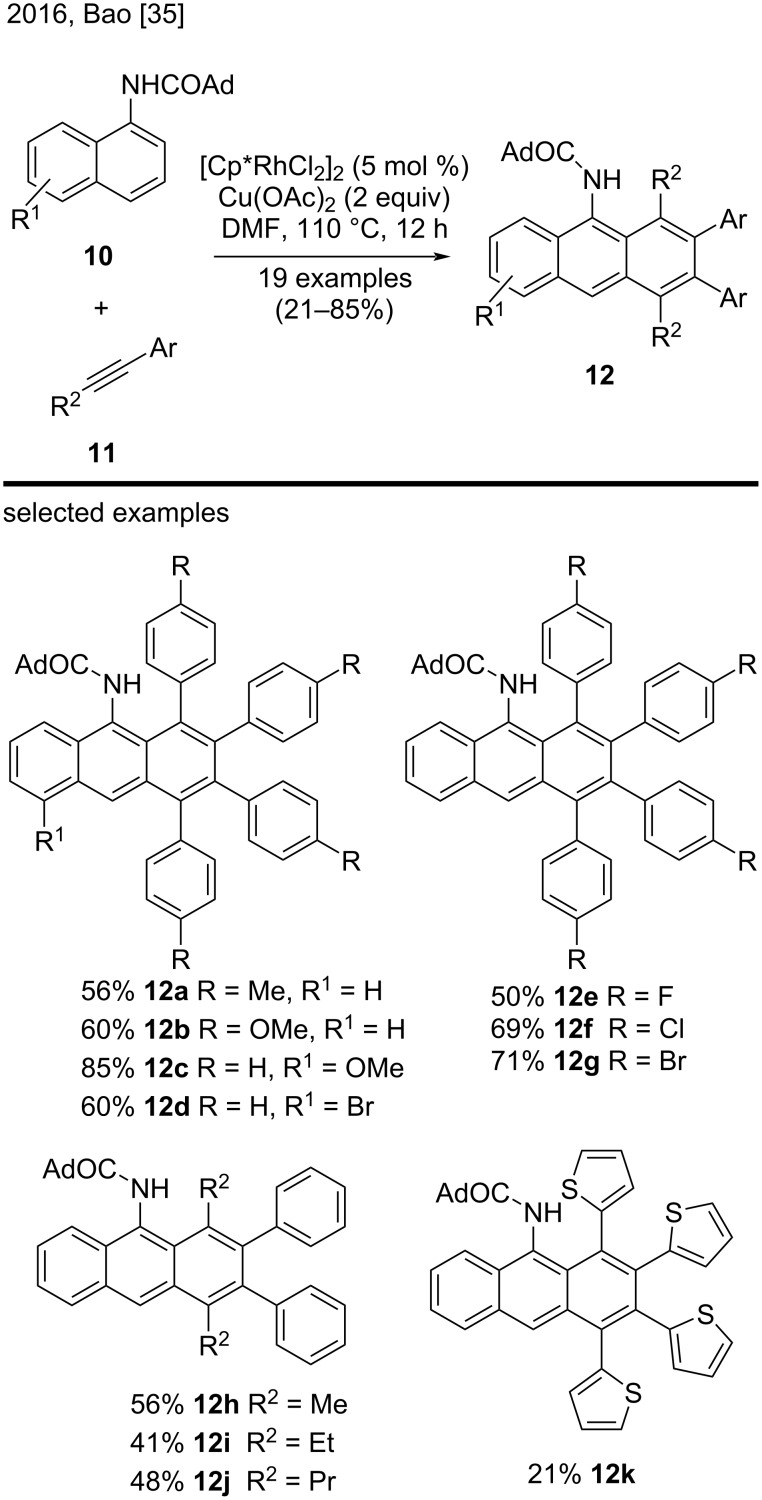
Rhodium-catalyzed oxidative benzannulation reactions of 1-adamantoyl-1-naphthylamines with internal alkynes.

A few years later, Bao et al. employed a similar approach to synthesize substituted anthracenes by the regioselective oxidative benzannulation of 1-adamantyl-1-naphthylamines **10** with internal alkynes **11** (Scheme 2) [35]. To the best of our knowledge, this was the first successful example of rhodium-catalyzed benzannulation reactions of *N*-adamantyl-1-naphthylamines. Reactions of **10** with internal alkynes **11** bearing an electron-donating or electron-withdrawing group on the benzene ring resulted in the corresponding substituted anthracenes **12** in moderate to good yields (see the representative examples **12a**–**g**). The same authors also investigated the applicability of reacting internal and asymmetric alkynes with heterocyclic compounds and obtained reasonable to satisfactory results (examples **12h**–**k**) [35]. Cu(OAc)_2_ proved to be an essential oxidant for the success of both the Miura and the Bao methodologies [34–35].

**Scheme 3:**
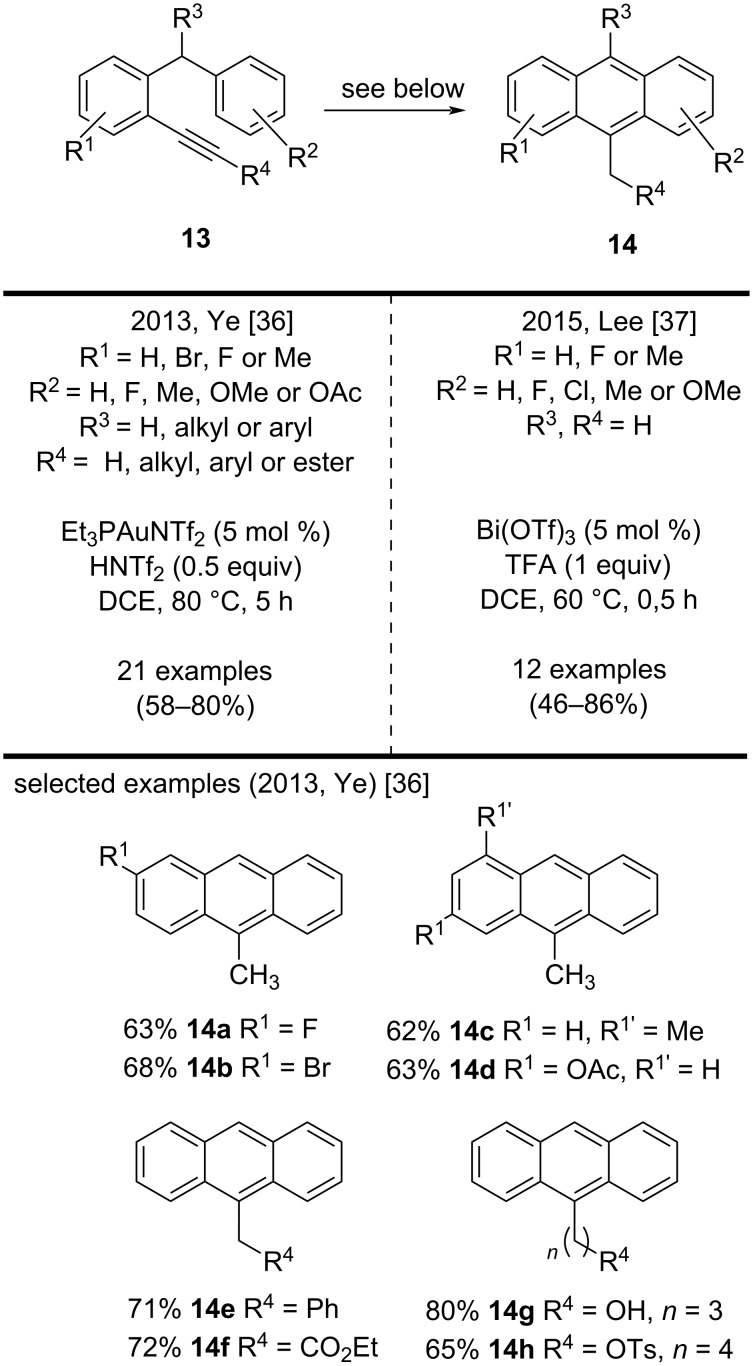
Gold/bismuth-catalyzed cyclization of *o*-alkynyldiarylmethanes.

In 2013, Ye and co-workers reported a concise method to synthesize substituted anthracenes **14** through a gold-catalyzed cyclization of *o*-alkynyldiarylmethanes **13** (Scheme 3) [36]. The scope of this reaction consisted of 21 examples in good yields (58–80%). Interestingly, the authors described that the F, Br, and Me functionalities and even the acid-sensitive OAc group on the aromatic ring were well tolerated during the cyclization, affording the corresponding anthracenes **14a**–**d**. The authors expanded the scope of the reaction to internal alkyne substrates and obtained the corresponding substituted anthracenes. The most representative examples included compounds **14e**–**h** [36]. In 2015, Lee and co-workers reported a similar approach. They published the synthesis of substituted 9-methylanthracenes **14** by cyclization of *o*-alkynyldiarylmethanes **13** in the presence of a bismuth catalyst. The scope of this reaction consisted of 12 examples in good yields (46–86%). They showed that the introduction of highly electronegative halides, such as fluorine or chlorine, on the phenyl ring afforded the substituted 9-methylanthracenes in lower yields. In addition, the method proposed by Lee and co-workers presented advantages that included shorter reaction times and milder reaction conditions [37].

**Scheme 4:**
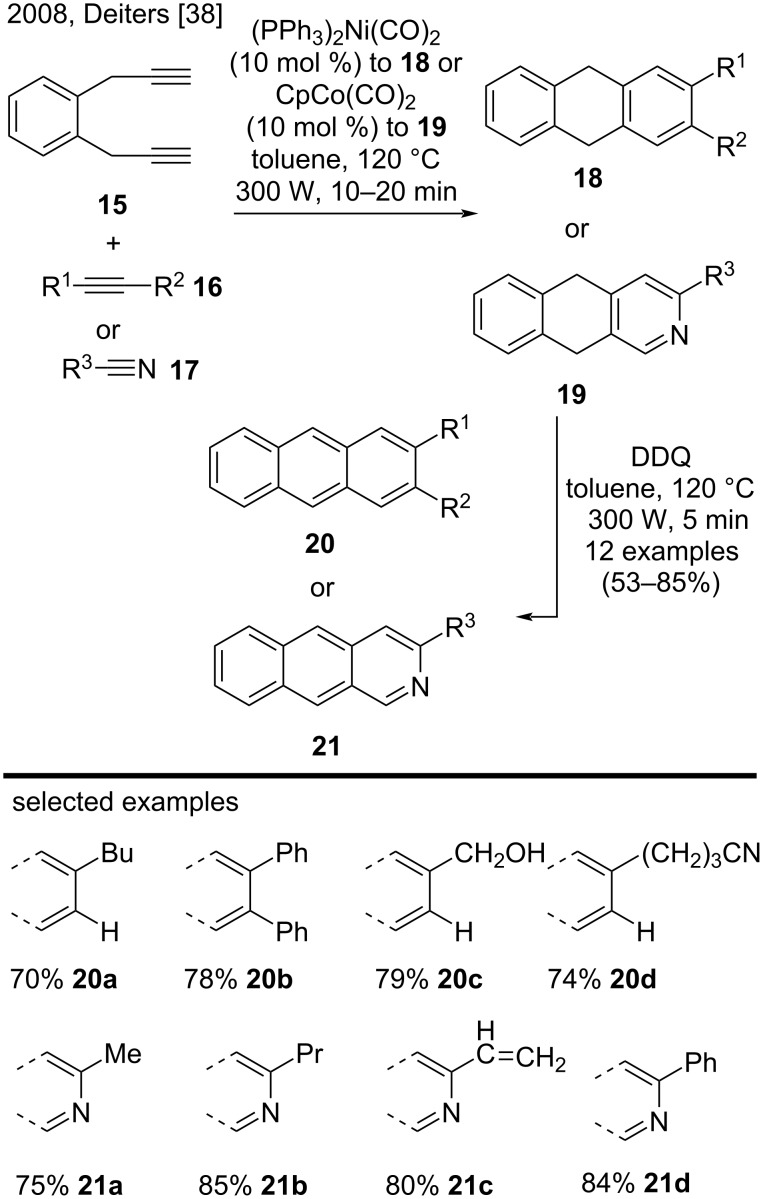
[2 + 2 + 2] Cyclotrimerization reactions with alkynes/nitriles in the presence of nickel and cobalt catalysts.

The direct synthesis of anthracene derivatives is rare and most methods usually involve more than one reaction step. For example, in 2008, Deiters and co-workers described an efficient two-step route to prepare substituted anthracenes and azaanthracenes via microwave-assisted [2 + 2 + 2] cyclotrimerization reactions in the presence of nickel and cobalt catalysts [38]. First, they employed diyne **15** in the reaction with a series of alkynes (**16**) or nitriles (**17**) bearing a variety of functional groups including alkyl and alkene chains, hydroxy groups, and benzene and pyridine rings, to achieve the corresponding cyclotrimerization products **18** or **19** (Scheme 4). The subsequent DDQ oxidation step yielded anthracenes **20** or azaanthracenes **21** in good yields (see the representative examples **20a**–**d** and **21a**–**d**) [38].

**Scheme 5:**
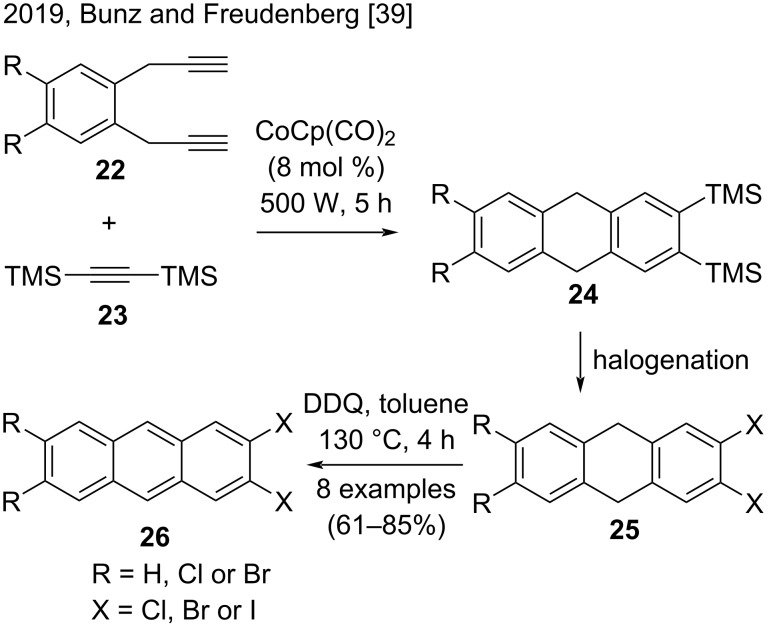
Cobalt-catalyzed [2 + 2 + 2] cyclotrimerization reactions with bis(trimethylsilyl)acetylene (**23**).

Recently, in a related approach, Bunz, Freudenberg, and co-workers described a useful route to obtain 2,3- and 2,3,6,7-halogenated anthracenes **26** by using CpCo(CO)_2_ as catalyst (Scheme 5) [39]. This synthesis started with a cobalt-catalyzed cyclotrimerization of previously prepared bis(propargyl)benzenes **22** and bis(trimethylsilyl)acetylene (**23**), affording the TMS-substituted cyclotrimerization products **24**. Next, the key step was introducing chlorine, bromine, or iodine substituents by halodesilylation of **24**. With the halogenated products **25** in hands, the authors employed DDQ in the oxidation/aromatization step, to obtain the di- and tetrahaloanthracenes **26** in good yields (61–85%) [39]. This methodology was notable for being an alternative method to synthesize 2,3,6,7-halogenated anthracene derivatives, which are difficult to obtain.

**Scheme 6:**
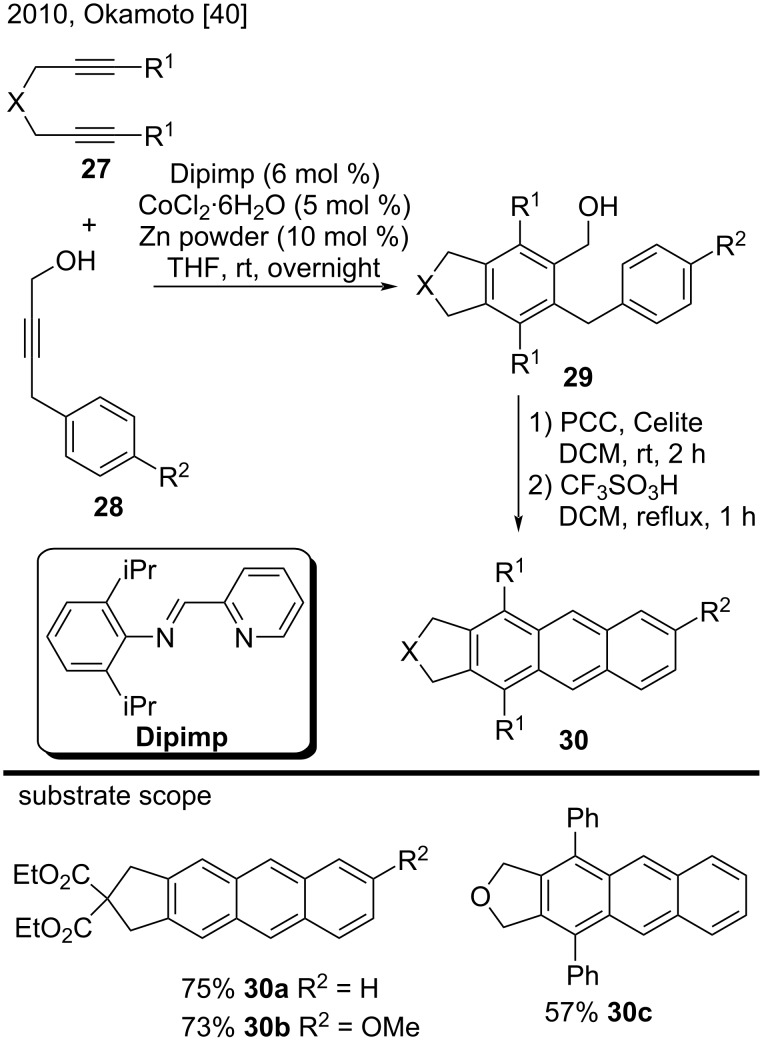
[2 + 2 + 2] Alkyne-cyclotrimerization reactions catalyzed by a CoCl_2_·6H_2_O/Zn reagent.

In 2010, Okamoto et al. published a three-step procedure to synthesize substituted anthracenes, pentaphenes, and trinaphthylenes via a [2 + 2 + 2] alkyne-cyclotrimerization reaction catalyzed by a cobalt/zinc reagent [40]. With regard to substituted anthracenes, this method consisted of a [2 + 2 + 2] cycloaddition reaction of 1,6-diynes **27** with 4-aryl-2-butyn-1-ols **28** (Scheme 6). The authors converted the resulting benzylic alcohols **29** to the corresponding aldehydes by treatment with PCC/Celite in dichloromethane (DCM). Finally, treatment with a catalytic amount of CF_3_SO_3_H provided the corresponding anthracenes **30a**–**c** in good yields (57–75%) [40].

#### Metal-catalyzed C–H bond activation

**Scheme 7:**
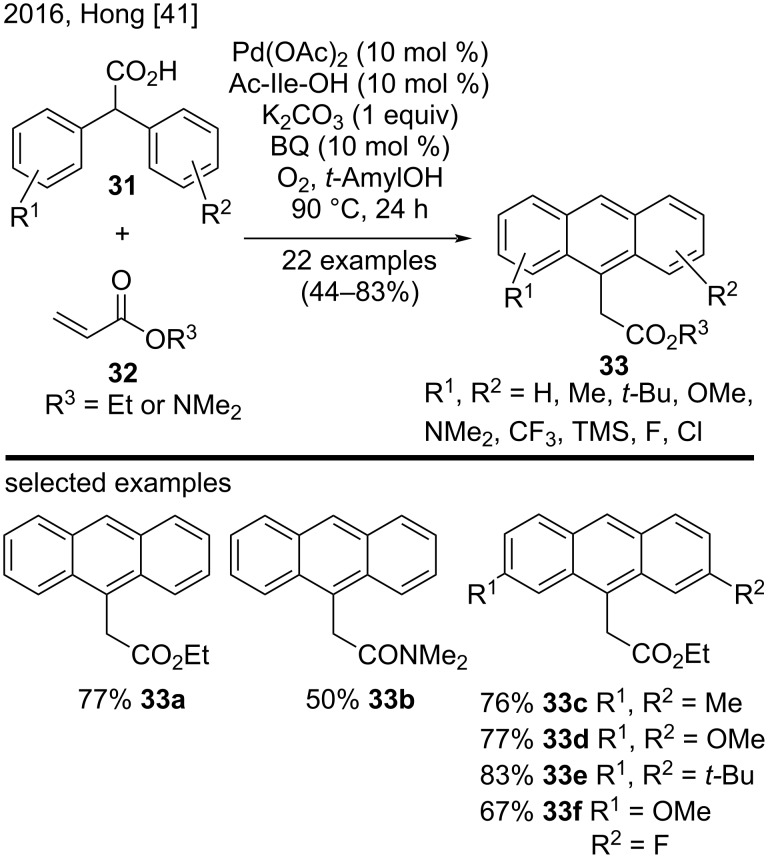
Pd(II)-catalyzed sp^3^ C–H alkenylation of diphenyl carboxylic acids with acrylates.

In 2016, Hong’s group developed a synthetic strategy to generate substituted anthracene derivatives **33**. The strategy involved a palladium(II)-catalyzed tandem transformation with diphenyl carboxylic acids **31** and acrylates **32** (Scheme 7) [41]. This new methodology involved a carboxyl-directed C–H alkenylation, a carboxyl-directed secondary C–H activation, an intramolecular C–C-bond formation, and further decarboxylative aromatization. The authors used several diphenyl carboxylic acids bearing electron-donating and electron-withdrawing groups on the aromatic rings to produce the corresponding substituted anthracenes, such as compounds **33a**–**f**, in good yields [41].

**Scheme 8:**
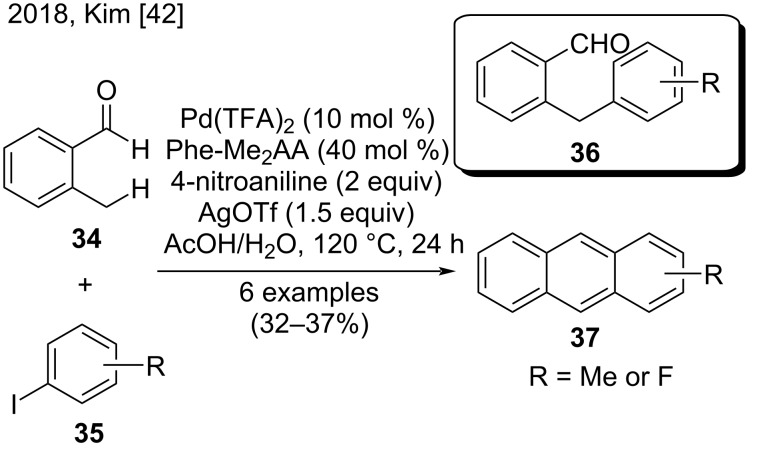
Pd(II)-catalyzed sp^3^ C–H arylation with *o*-tolualdehydes and aryl iodides.

Recently, Kim and co-workers reported a one-pot synthesis of substituted anthracenes **37** from *o*-tolualdehyde **34** and aryl iodides **35** via a palladium-catalyzed C–H arylation with a silver oxidant (Scheme 8) [42]. During optimization studies, the authors noted that steric and electronic effects strongly affected the cyclization generating the anthracenes. For example, reactions with *o*-tolualdehydes bearing electron-withdrawing substituents showed poor conversions. In addition, by simply changing AgTFA to AgOTf allowed anthracenes **37** to be obtained in low yields (32–37%) instead of arylated products **36**, the latter of which were more efficiently cyclized [42].

#### Friedel–Crafts alkylation of arenes with aromatic aldehydes

**Scheme 9:**
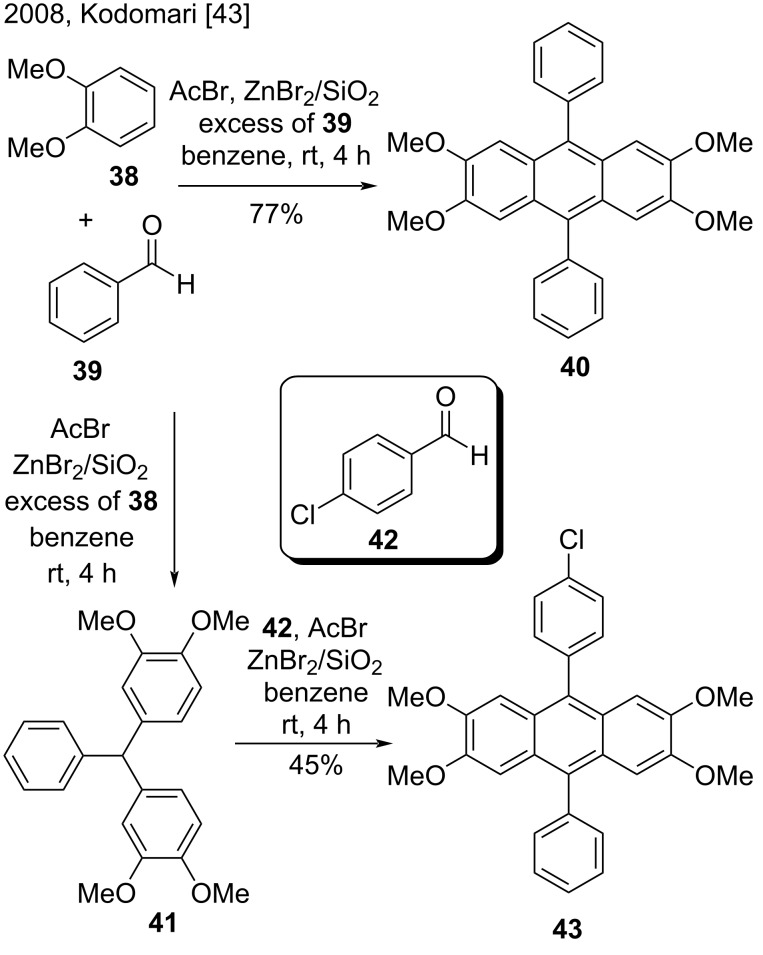
Alkylation of arenes with aromatic aldehydes in the presence of acetyl bromide and ZnBr_2_/SiO_2_.

The Lewis acid-catalyzed Friedel–Crafts alkylation of electron-rich arenes with aromatic aldehydes has proven an efficient and often direct method to prepare anthracene derivatives. Kodomari and co-workers disclosed a convenient synthesis of triarylmethanes and 9,10-diarylanthracenes from reactions of arenes and aromatic aldehydes by using acetyl bromide in the presence of silica gel-supported zinc bromide [43]. The methodology developed by these authors involved using excess of one of the reagents. When the authors employed excess benzaldehyde (**39**) in the reaction with veratrole (**38**), they obtained 9,10-diarylanthracene **40** in good yield (Scheme 9). On the other hand, the reaction of excess veratrole (**38**) with benzaldehyde (**39**) produced triarylmethane **41**. The reaction of **41** with 4-chlorobenzaldehyde (**42**) afforded 9,10-diarylanthracene **43**, so the authors concluded that triarylmethane is an intermediate in the reaction with excess benzaldehyde [43].

**Scheme 10:**
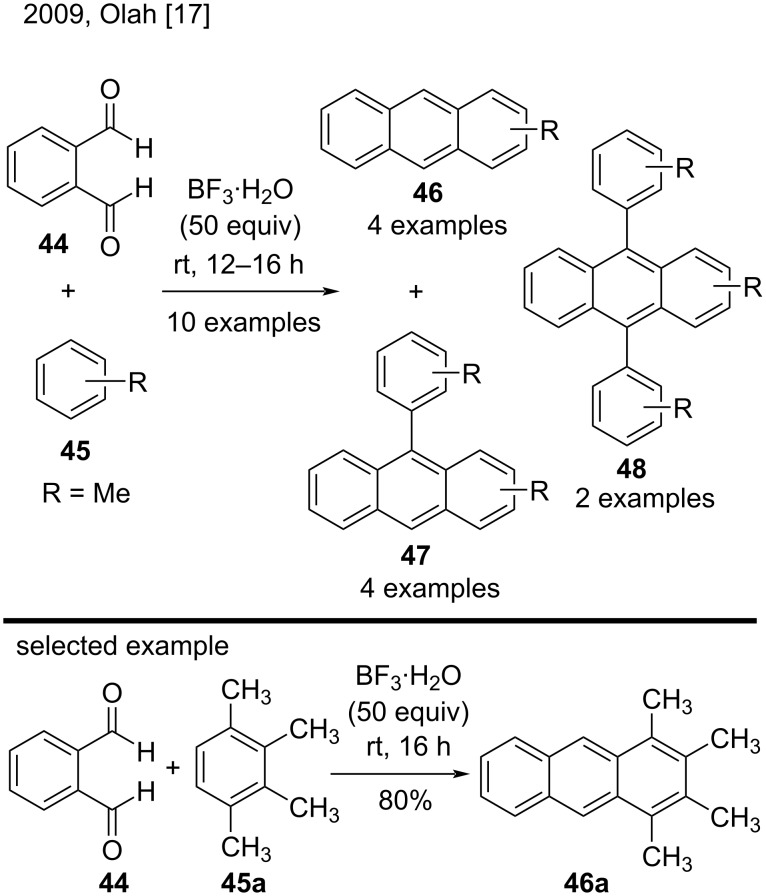
BF_3_·H_2_O-catalyzed hydroxyalkylation of arenes with aromatic dialdehyde **44**.

In 2009, Olah’s group applied BF_3_ monohydrate as acid catalyst in arene hydroxyalkylation with aromatic aldehydes, to provide triarylmethane, diarylmethylbenzaldehyde, and anthracene derivatives. In this work, the reaction of phthalaldehyde (**44**) with arenes **45** resulted in anthracene derivatives **46**–**48** as major products (Scheme 10) [17]. In fact, the authors obtained mixtures of anthracene derivatives, with 9-arylanthracenes **47** in greater proportion in almost all reactions. On the other hand, the reaction of **44** with 1,2,3,4-tetramethylbenzene (**45a**) resulted in tetrametylanthracene **46a** in good yield (80%). However, the reaction only gave good results with electron-rich arenes [17].

**Scheme 11:**
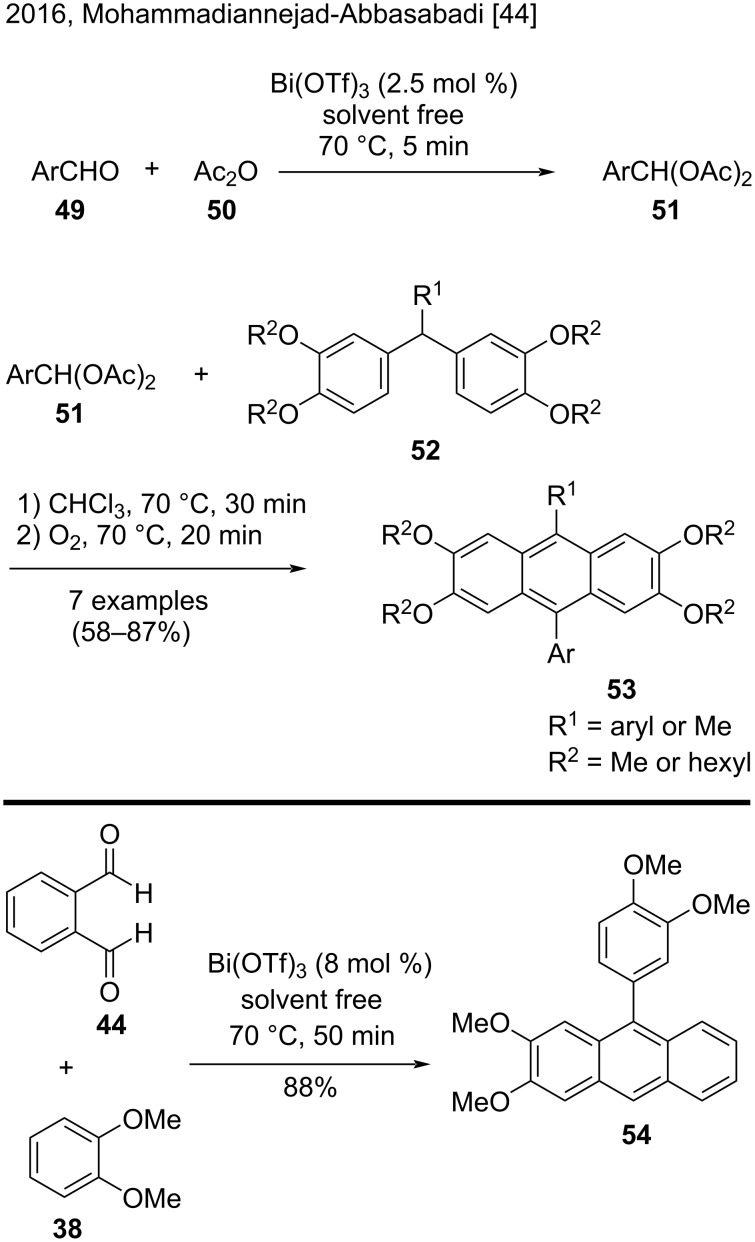
Bi(OTf)_3_-promoted Friedel–Crafts alkylation of triarylmethanes and aromatic acylals and of arenes and aromatic aldehydes.

In 2016, Mohammadiannejad-Abbasabadi and co-workers unveiled a three-step procedure to synthesize 9,10-disubstituted-anthracenes from bis(dihexyloxyphenyl)arylmethanes or diveratrylmethanes and aromatic acylals [44]. In the first step, reaction between aromatic aldehydes **49** and acetic anhydride (**50**) promoted by Bi(OTf)_3_ under solvent-free conditions afforded aromatic acylals **51** (Scheme 11). In the next two steps, the authors added previously prepared triarylmethanes **52** to the reaction mixture under air atmosphere, and then under oxygen atmosphere. Therefore, an efficient Bi(OTf)_3_/O_2_ system promoted the oxidation/aromatization step, providing the 9,10-disubstituted 2,3,6,7-tetraalkoxyanthracenes **53** in good yields (58–87%). Additionally, the authors reacted veratrole (**38**) and phthalaldehyde (**44**) in the presence of Bi(OTf)_3_, to obtain the substituted anthracene derivative **54** in good yield (88%). Under the same conditions, the reaction of veratrole (**38**) and isophthalaldehyde afforded only the corresponding triarylmethane in 93% yield, indicating that this reaction strongly depended on the nature of the aromatic aldehyde [44].

#### Synthesis of substituted anthracenes from anthraquinones

**Scheme 12:**
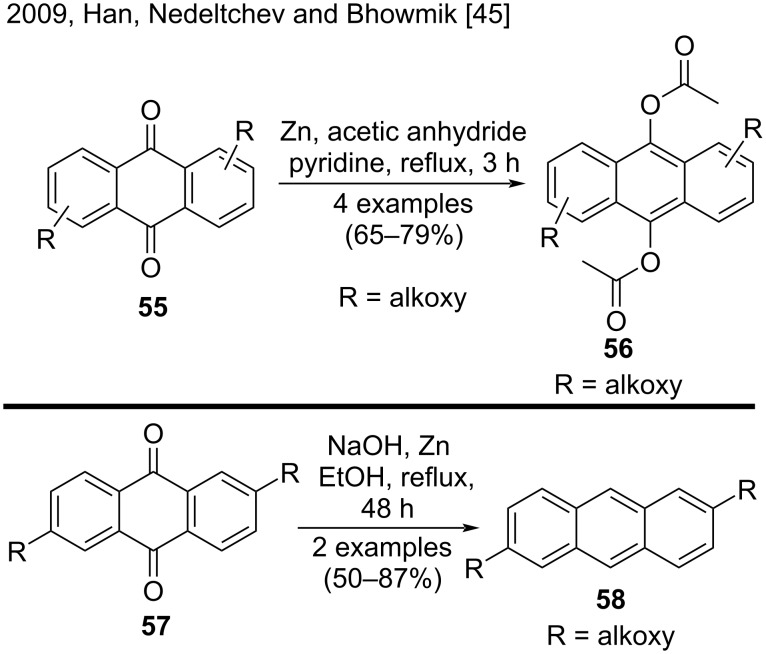
Reduction of anthraquinones by using Zn/pyridine or Zn/NaOH reductive methods.

An easy and common method to obtain anthracenes is to reduce anthraquinones by using several reagents. An important advantage of this method is that the reactive positions 9 and 10 of anthracene are protected, directing substitution to one of the other rings. By using this kind of methodology, in 2009, Han, Nedeltchev, and Bhowmik reported the facile synthesis of 9,10-diacetoxyanthracenes **56** and 2,6-dialkoxyanthracenes **58** from the corresponding anthraquinones **55** and **57** via a single-step reduction with either zinc/pyridine or zinc/NaOH (Scheme 12). The scope of their work consisted of six examples, and they obtained anthracene derivatives in moderate to good yields (50–87%) [45].

**Scheme 13:**
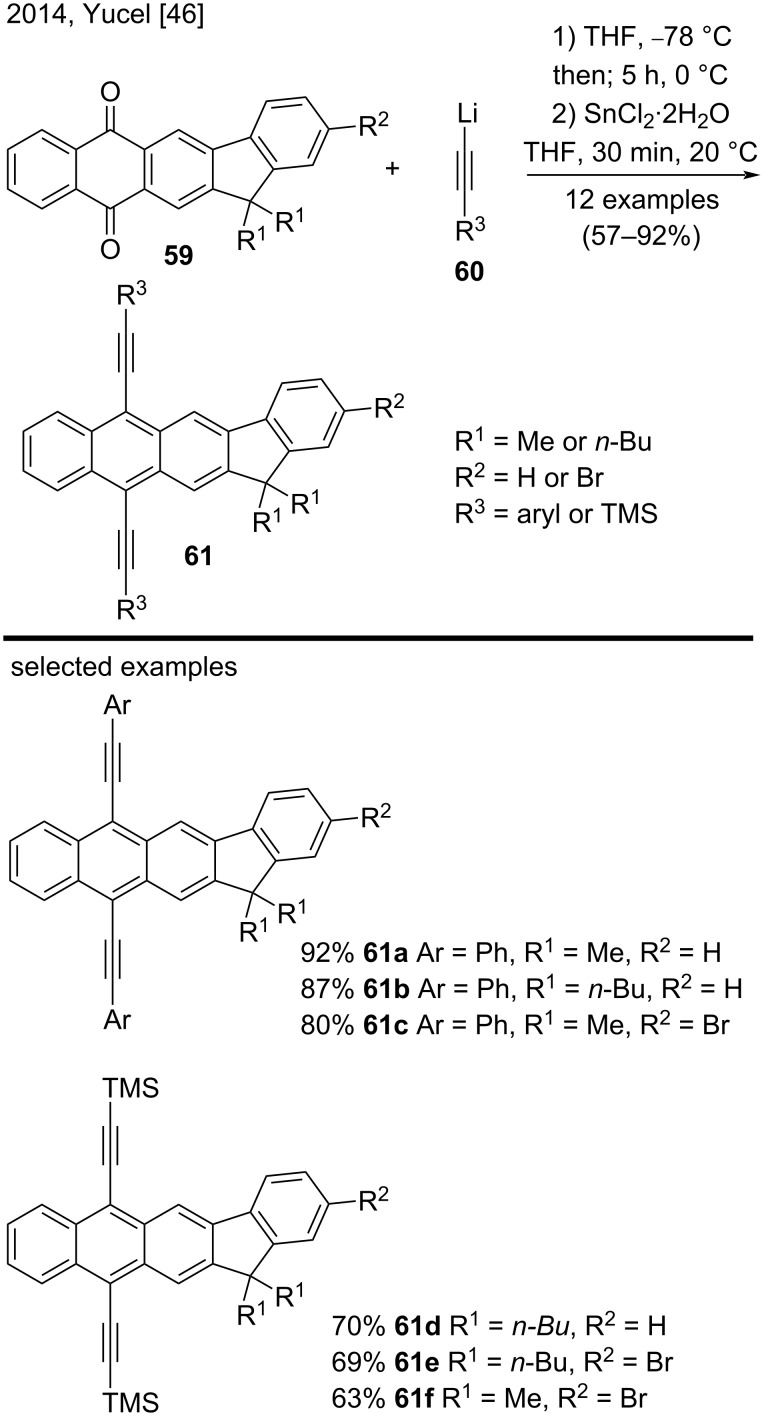
Two-step route to novel substituted Indenoanthracenes.

In 2014, Yucel and co-workers synthesized 12 novel indenoanthracene derivatives to study their optical, electrochemical, and thermal properties [46]. The authors prepared dialkynyl-substituted indenoanthracenes **61**, containing alkyl or bromine substituents, from the corresponding indenoantraquinones **59** in two steps in good to excellent yields (57–92%) (Scheme 13). Representative examples included indenoanthracenes **61a**–**c**, bearing aryl groups linked to the alkyne, and indenoanthracenes **61d**–**f**, containing tetramethylsilane groups at the terminal alkyne [46].

**Scheme 14:**
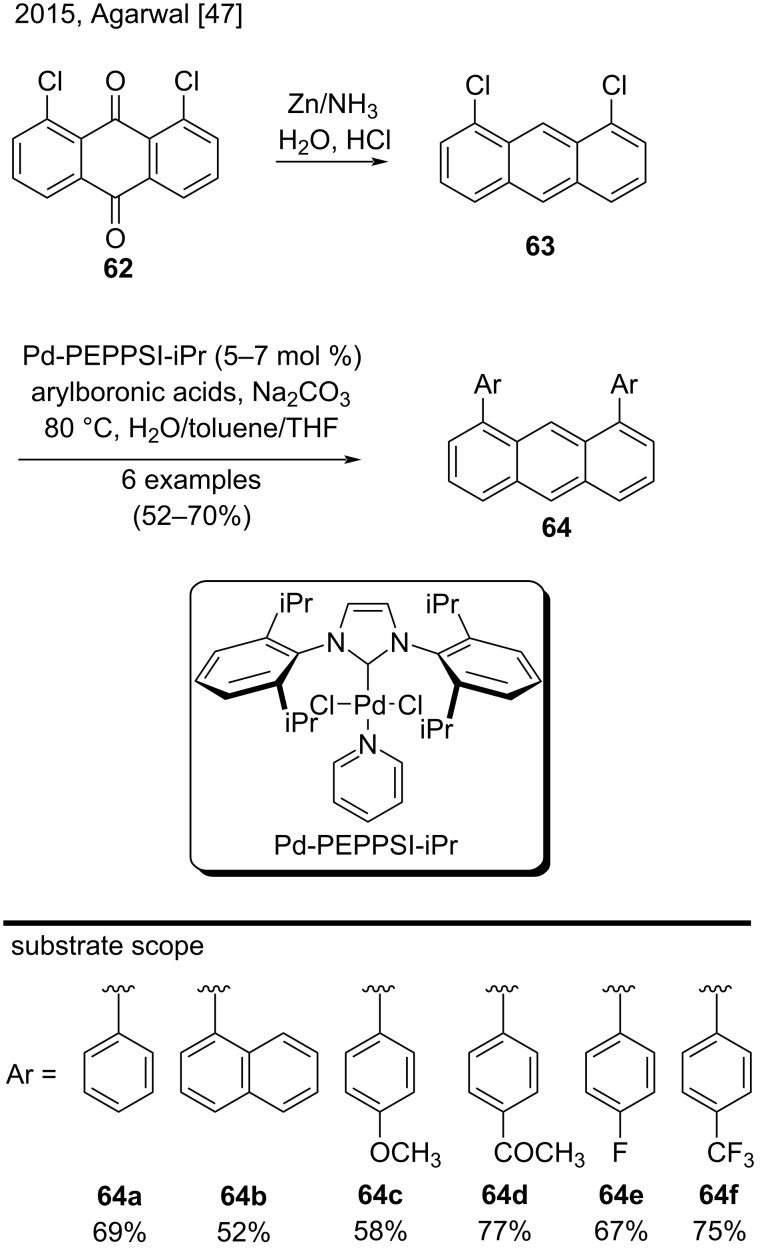
Synthesis of 1,8-diarylanthracenes through Suzuki–Miyaura coupling reaction in the presence of Pd-PEPPSI-iPr as catalyst.

From commercially available 1,8-dichloroanthraquinone (**62**) and by using modified Suzuki–Miyaura coupling reaction conditions, Agarwal et al. synthesized a series of 1,8-diarylanthracene derivatives **64** in two steps (Scheme 14) [47]. First, the simple reduction of anthraquinone **62** employing the well-known reductive Zn/NH_3_ system and HCl provided 1,8-dichloroanthracene (**63**). A Suzuki–Miyaura coupling reaction in the presence of Pd(PPh_3_)_4_ as catalyst, was inefficient with the chloro-substituted aryl substrates. However, the use of Pd-PEPPSI-iPr as catalyst solved this problem. By using this catalyst, the authors obtained 1,8-diarylanthracenes **64a**–**f** in good yields (52–77%) from reactions of the corresponding arylboronic acids [47].

**Scheme 15:**
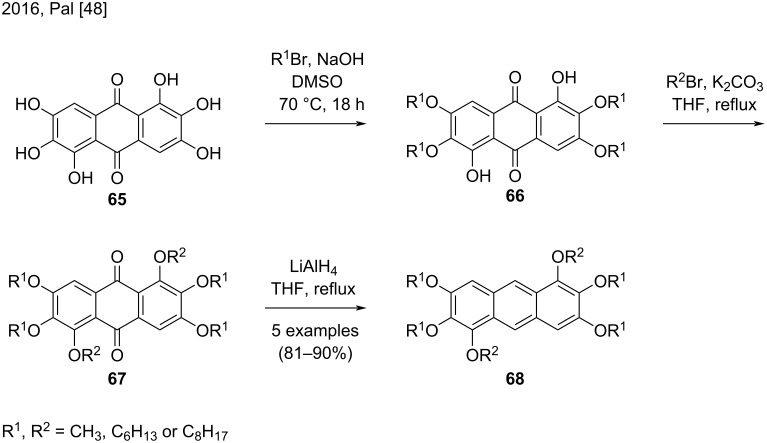
Synthesis of five new substituted anthracenes by using LAH as reducing agent.

In 2016, Pal and co-workers prepared five new substituted anthracene derivatives **68** containing six alkyl chains (Scheme 15) [48]. The authors synthesized hexahydroxyanthraquinone **65** and modified the functional groups in compound **65** and its derivative **66** according to reported methods. Although other methodologies were available, the authors chose to employ lithium aluminum hydride (LAH) to reduce the substituted anthraquinones **67** to the corresponding anthracenes **68**, to obtain very good yields (81–90%) [48].

**Scheme 16:**
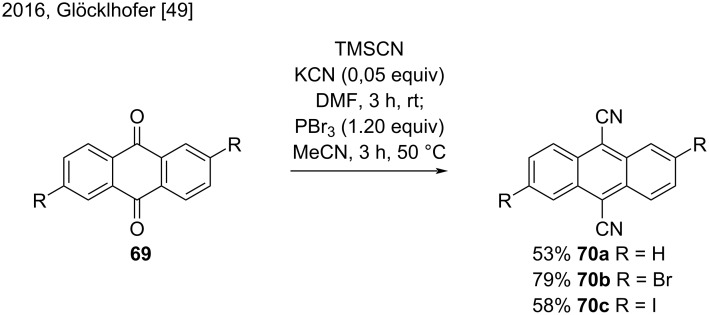
One-pot procedure to synthesize substituted 9,10-dicyanoanthracenes.

In 2016, Glöcklhofer and co-workers developed a versatile one-pot procedure for the direct synthesis of 9,10-dicyanoanthracenes from 9,10-anthraquinones [49]. Despite the challenges presented by the different substrates, a long study of reaction optimization allowed the authors to synthesize 9,10-dicyanoanthracene (**70a**), 2,6-dibromo- (**70b**), and 2,6-diiodo-9,10-dicyanoanthracene (**70c**) from the corresponding 9,10-anthraquinones **69** in good yields (53–79%) (Scheme 16) [49].

**Scheme 17:**
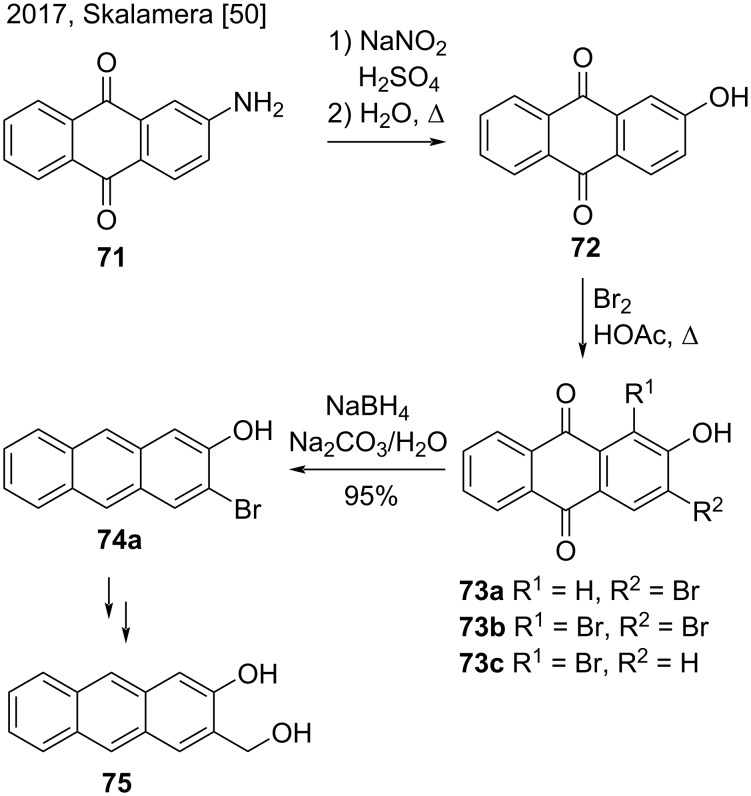
Reduction of bromoanthraquinones with NaBH_4_ in alkaline medium.

In 2017, Skalamera and co-workers reported a new synthetic pathway to produce 2,3-disubstituted anthracenes by functionalizing the corresponding anthraquinone and subsequent reduction with NaBH_4_ [50]. Via a known procedure, the authors converted commercially available 2-aminoanthraquinone (**71**) to 2-hydroxyanthraquinone (**72**), followed by bromination that led to a mixture of bromoanthraquinones **73a**–**c** (Scheme 17). According to the authors, many existing methods to reduce anthraquinones **73a** and **73b** have been tested, but only the one-step method with sodium borohydride in alkaline medium resulted in 2,3-disubstituted anthracene **74a**, the precursor of anthracene target **75**, in excellent yield (95%) [50].

#### Intramolecular cyclization

Intramolecular cyclization is a broad category that covers several known methodologies for the synthesis of anthracene derivatives. Traditionally, widely used methods include acid-catalyzed Friedel–Crafts intramolecular cyclization and Bradsher-type reactions and their variations [21]. Interestingly, some of the methods already reviewed here could also be included in this category although they are classified in other categories that are also appropriate.

**Scheme 18:**
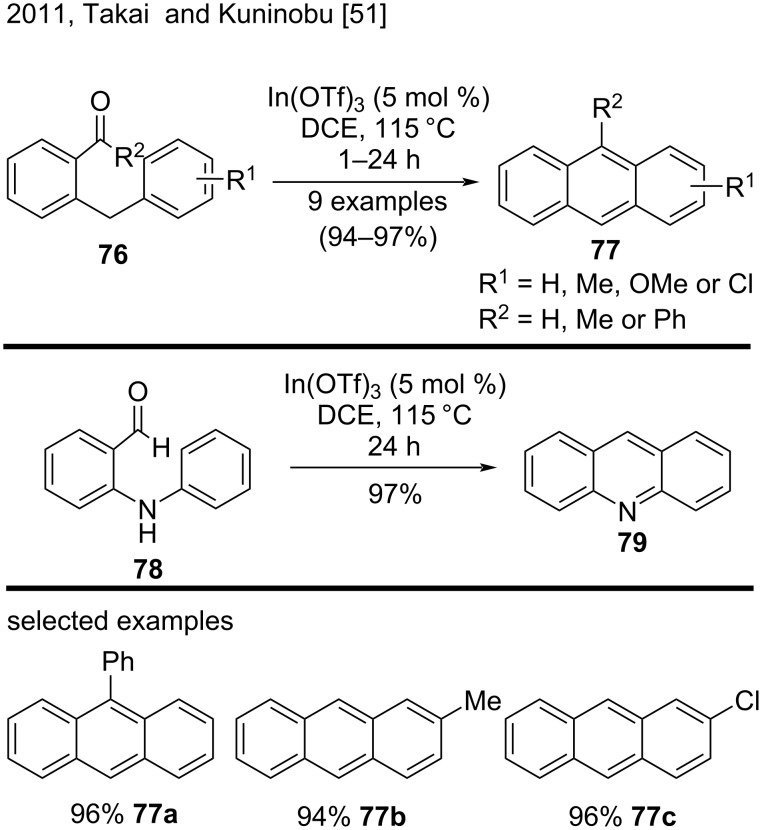
In(III)-catalyzed reductive-dehydration intramolecular cycloaromatization of 2-benzylic aromatic aldehydes/ketones.

Takai, Kuninobu, and their research group used an indium catalyst to synthesize polycyclic aromatic compounds via a reductive-dehydration intramolecular cycloaromatization [51], which is a Bradsher-type reaction. The authors prepared substituted anthracenes **77**, bearing phenyl, methyl, chloro, or methoxy groups, such as **77a**–**c** in excellent yields (94–96%) by treating the corresponding 2-benzylic aromatic aldehydes/ketones **76** with a catalytic amount of In(OTf)_3_ (Scheme 18) [51]. In addition, the authors explored this methodology to obtain polycyclic aza-aromatic compounds. They achieved promising results and obtained the aza-analogue acridine (**79**) from 2-(phenylamino)benzaldehyde (**78**) in excellent yield (97%) [51].

**Scheme 19:**
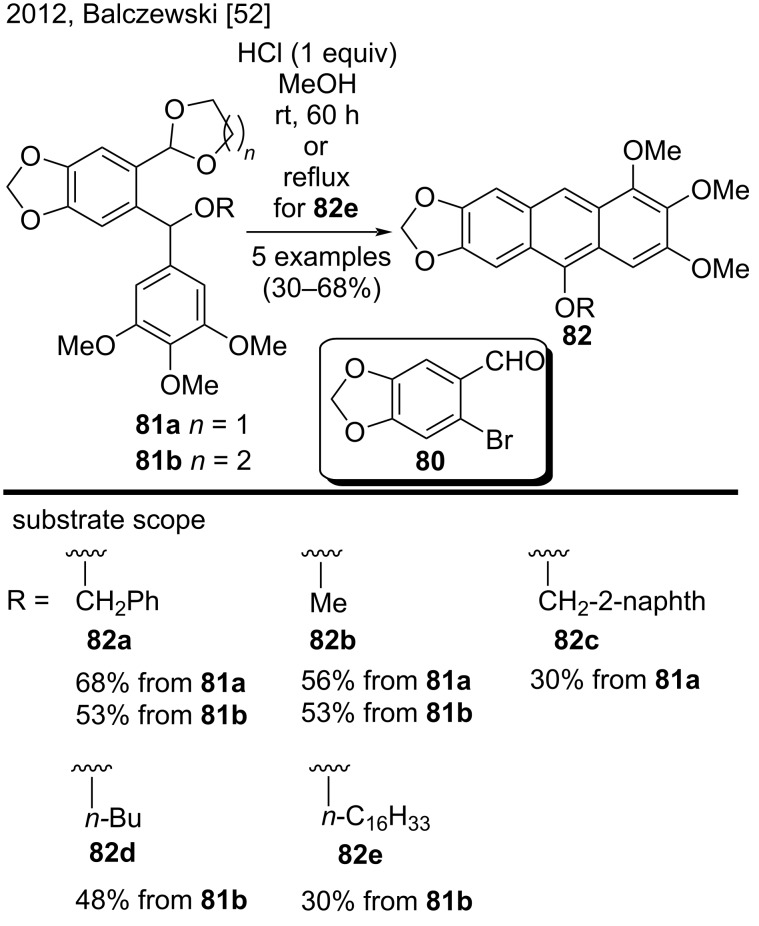
Acid-catalyzed cyclization of new *O*-protected *ortho*-acetal diarylmethanols.

In 2012, Balczewski and co-workers reported a new methodology to synthesize 10-OR-substituted anthracenes **82** via an acid-catalyzed cyclization of *O*-protected *ortho*-acetal diarylmethanols **81a** and **81b** as a new type of reactants (Scheme 19) [52]. To carry out the cyclization step, this new methodology employed a diluted aqueous methanolic solution of HCl at room temperature. This step was based on a modified intramolecular Friedel–Crafts-type cyclization. This was the first report of the same molecule bearing an acid-sensitive acetal and dibenzyl alkoxy groups. The key steps described in the work were protection of the aldehyde group of 6-bromopiperonal (**80**) by using 1,2-ethanediol or 1,3-propanediol, followed by sequential transformations of the resulting adduct into the protected diarylmethanols **81a** and **81b**. The scope of the reaction consisted of five examples (**82a**–**e**) that were obtained in moderate yields (30–68%). According to the authors, the reaction conditions were the mildest ever used in this type of intramolecular cyclization until 2012 [52].

**Scheme 20:**
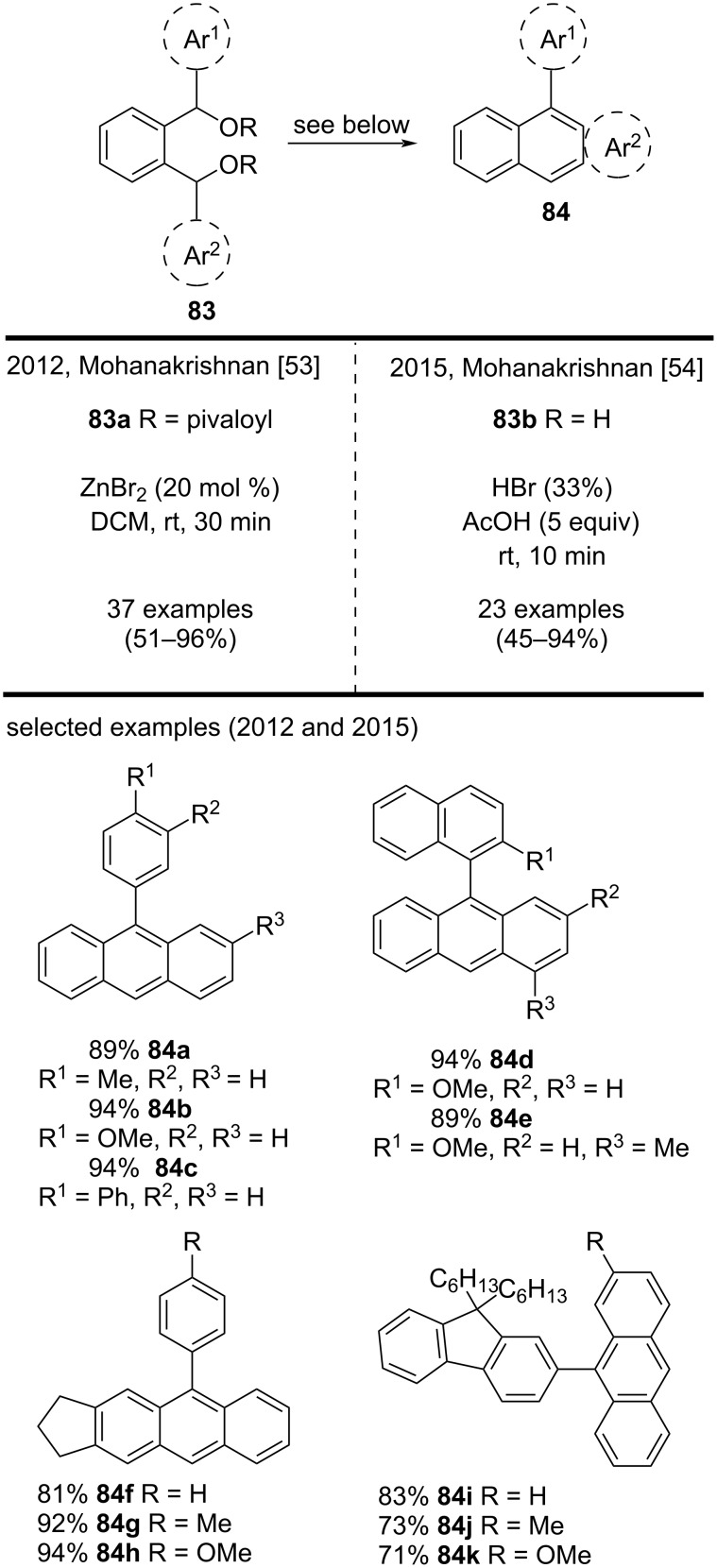
Lewis acid-mediated regioselective cyclization of asymmetric diarylmethine dipivalates and diarylmethine diols.

Mohanakrishnan’s group has contributed with numerous methodologies for the synthesis of anthracene derivatives, mainly methodologies involving Lewis acid-mediated intramolecular cyclizations. For example, in 2012 they reported an annulation protocol to synthesize anthracene, tetracene, and naphtho[*b*]thiophene derivatives via ZnBr_2_-mediated regioselective annulation of asymmetric 1,2-diarylmethine dipivalates **83a** (Scheme 20). On the basis of this methodology, they prepared 37 examples of different types of anthracene derivatives, such as compounds **84a**–**e**, in very good yields (89–94%) and mild reaction conditions [53].

**Scheme 21:**
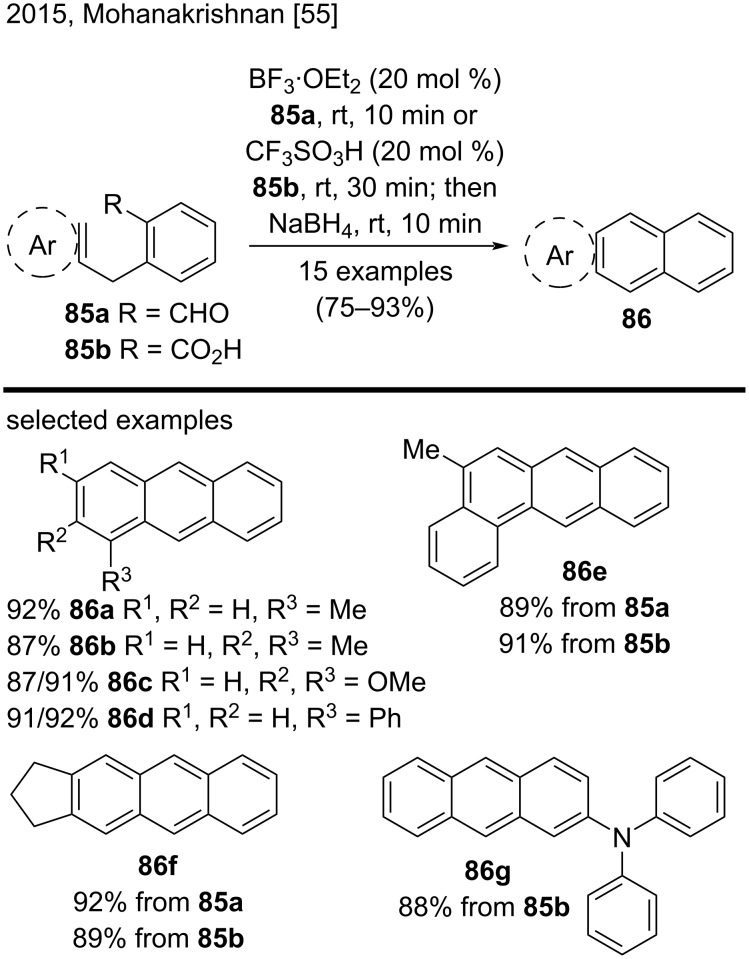
BF_3_·OEt_2_/CF_3_SO_3_H-mediated cyclodehydration reactions of 2-(arylmethyl)benzaldehydes and 2-(arylmethyl)benzoic acids.

In a related approach, in 2015, Mohanakrishnan and his group reported the synthesis of anthracene derivatives and other annulated products via the regioselective cyclization of asymmetric 1,2-diarylmethine diol **83b** by using a HBr/AcOH system (Scheme 20) [54]. They obtained substituted anthracene derivatives in very good yields (71–94%) and representative examples included **84f**–**k** [54]. By using both methodologies, the authors obtained substituted benzo[*a*]anthracenes from compounds **83**. In 2015, Mohanakrishnan’s group also disclosed a Bradsher-type cyclodehydration of substituted 2-arylmethylbenzaldehyde **85a** mediated by BF_3_·OEt_2_ (Scheme 21) [55]. By using this methodology, they prepared substituted anthracene derivatives **86** in excellent yields (75–93%). Impressively, the authors were able to synthesize these compounds in better yields via a two-step procedure involving the cyclization of 2-arylmethylbenzoic acid **85b** with triflic acid, followed by a NaBH_4_-mediated reductive dehydration. Some representative examples included anthracenes **86a**–**g**. For those interested, the cyclodehydration was also extended to 2-(arylmethyl)naphthaldehydes and 2-(arylmethyl)naphthoic acids, to produce the corresponding annulated compounds [55].

**Scheme 22:**
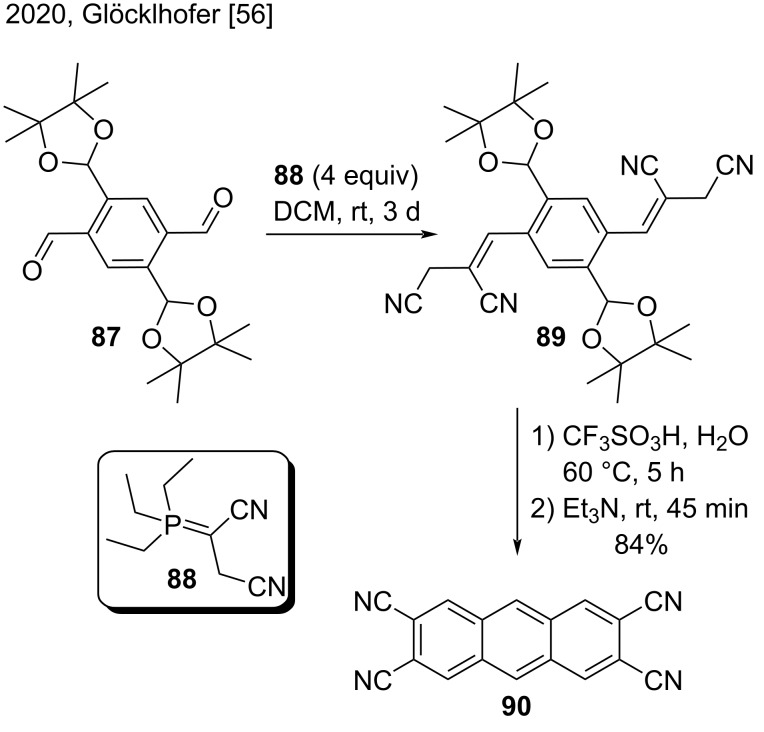
Synthesis of 2,3,6,7-anthracenetetracarbonitrile (**90**) by double Wittig reaction followed by deprotection and intramolecular double ring-closing reaction.

Recently, Glöcklhofer and co-workers developed a promising method to prepare 2,3,6,7-substituted anthracene derivatives via an intramolecular double ring-closing condensation approach (Scheme 22) [56]. As a demonstration of this new methodology, they synthesized 2,3,6,7-anthracenetetracarbonitrile (**90**) in a good yield (84%) by double intermolecular Wittig reaction of the protected benzenetetracarbaldehyde **87** with reagent **88**, followed by deprotection and double ring-closing reaction of intermediate **89**; they used a mixture of triflic acid/water in the first step and trimethylamine in the next reaction step [56].

#### Other procedures

**Scheme 23:**
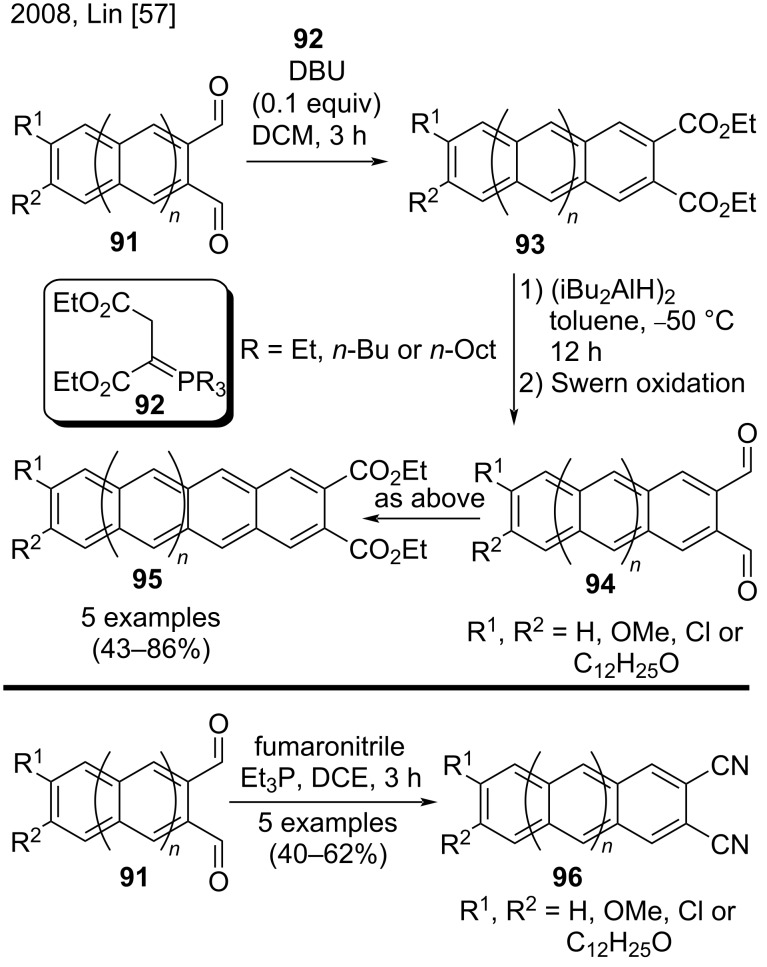
Homo-elongation protocol for the synthesis of substituted acene diesters/dinitriles.

In 2008, Lin et al. reported a homo-elongation protocol to obtain acene diesters and dinitriles starting from dialdehydes (Scheme 23) [57]. This methodology was based on a Wittig reaction between substituted [*n*]acene-2,3-dicarbaldehydes **91** and the Wittig reagents **92**; DBU was employed to produce the corresponding substituted [*n*+1]acene-2,3-diethyl diesters **93**. Then, in two steps (reduction and Swern oxidation), the authors converted the diesters **93** to the dialdehydes **94**, which could be transformed into the corresponding [*n*+2]acene-2,3-diethyl diesters **95** by the procedure described above. The scope of the reaction included five examples of substituted anthracene-2,3-dicarboxylates bearing Cl or alkoxy groups (43–86% yield). Lin et al. also applied the same approach to synthesize acene-2,3-dinitriles **96** by using fumaronitrile to produce the Wittig reagents. Despite the limitations, the authors noted that DBU was no longer required in these reactions. The scope of these reactions included five examples of substituted anthracene-2,3-dicarbonitriles **96** (40–62% yield) [57].

**Scheme 24:**
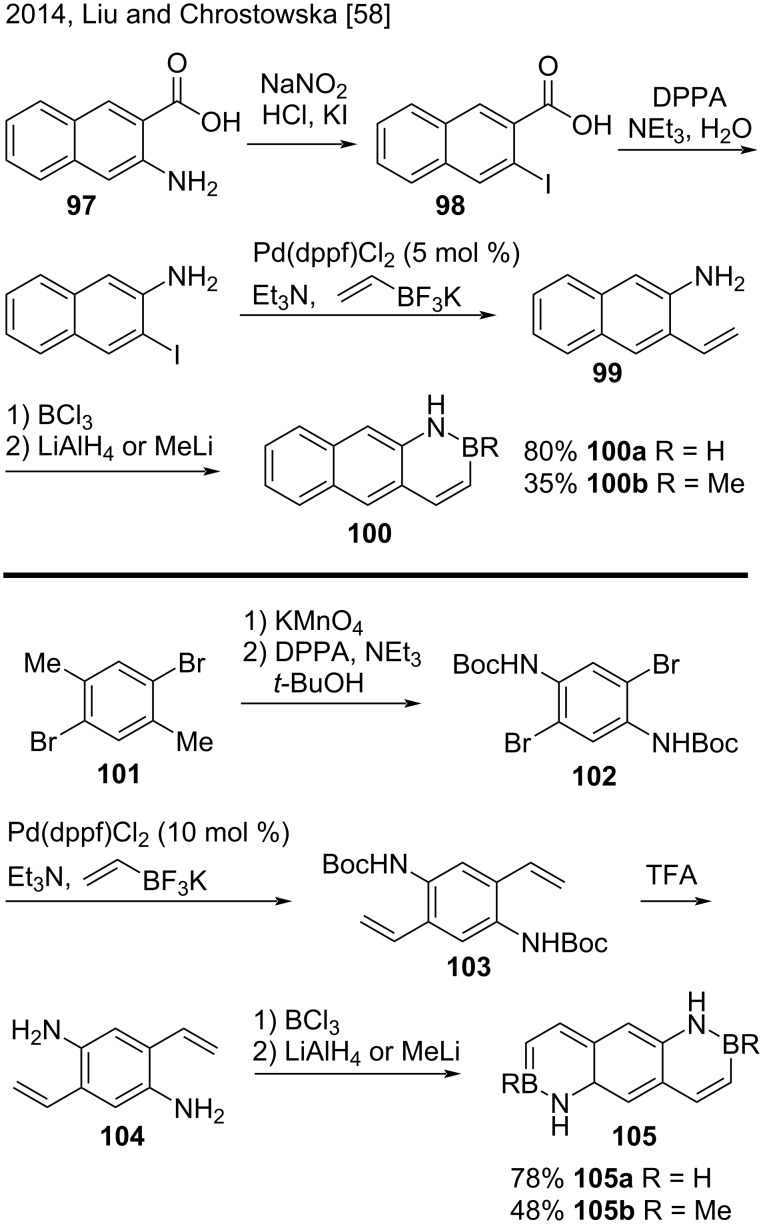
Synthesis of two new parental BN anthracenes via borylative cyclization.

BN arenes include analogs in which a C=C bond has been replaced with an isoelectronic BN bond. In 2014, Liu, Chrostowska, and co-workers synthesized two new parental BN anthracenes **100** and **105** (Scheme 24) [58]. The proposed method consisted of an adaptation of Dewar’s original protocol to prepare 1,2-BN naphthalenes [58]. First, the authors submitted 3-amino-2-naphthoic acid (**97**) to a Sandmeyer reaction and obtained **98**. Then, a Curtius rearrangement of **98** by using diphenylphosphoryl azide (DPPA) yielded 3-iodonaphthalen-2-amine, that was subjected to a Suzuki cross-coupling with potassium vinyltrifluoroborate resulting in aminostyrene **99**. The key step was borylative cyclization of precursor **99** and the subsequent treatment with LiAlH_4_ or MeLi, which resulted in BN anthracene **100a** or **100b**. On the other hand, the authors obtained BN anthracenes **105a** and **105b** by oxidation followed by Curtis rearrangement of the commercially available 1,4-dibromo-2,5-dimethylbenzene (**101**), with subsequent Suzuki cross-coupling of compound **102** with potassium vinyltrifluoroborate, removal of the *N*-Boc protecting group of **103** with trifluoroacetic acid, and borylative cyclization of precursor aminostyrene **104** [58].

**Scheme 25:**
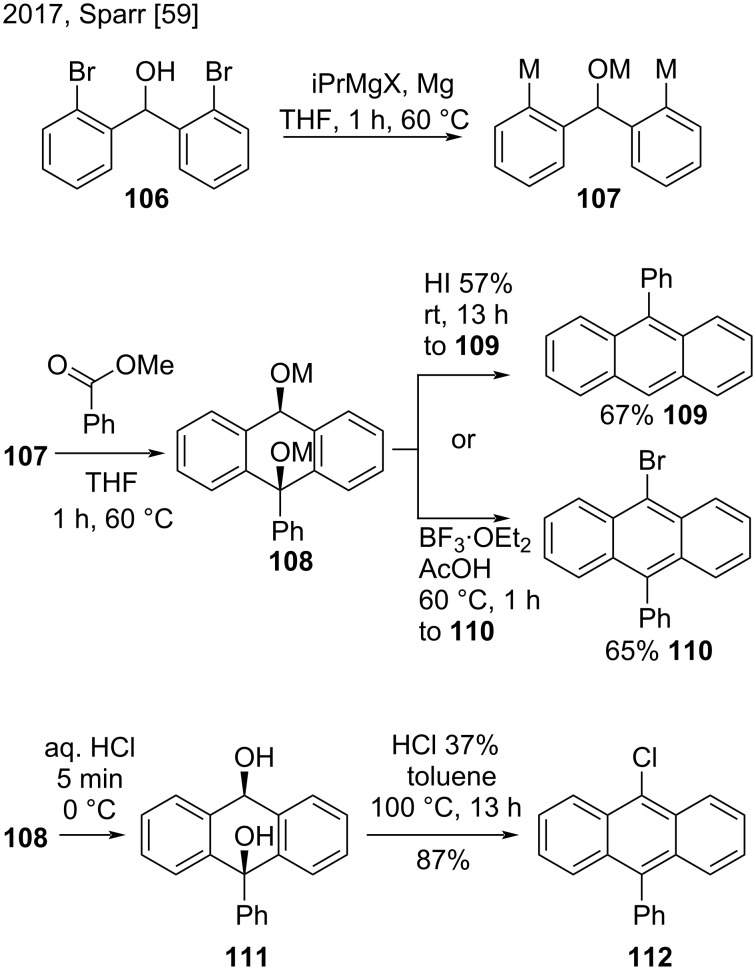
Synthesis of substituted anthracenes from a bifunctional organomagnesium alkoxide.

Sparr’s research group developed the 1,5-bifunctional organomagnesium alkoxide reagent **107**, which converted esters into di- and monofunctionalized anthracenes (Scheme 25) [59]. They prepared this reagent by deprotonation–magnesiation of compound **106**. Then, the treatment of aromatic esters with **107** produced dialkoxide **108**, which could be easily converted to the substituted anthracenes **109** and **110** by varying the acidic workup procedures. In addition, they prepared 9-chloro-10-phenylanthracene (**112**) in good yield (87%) through diol **111** [59].

### Synthesis of substituted benzo[*a*]anthracene and dibenzoanthracene derivatives

#### Metal-catalyzed C–H bond activation

**Scheme 26:**
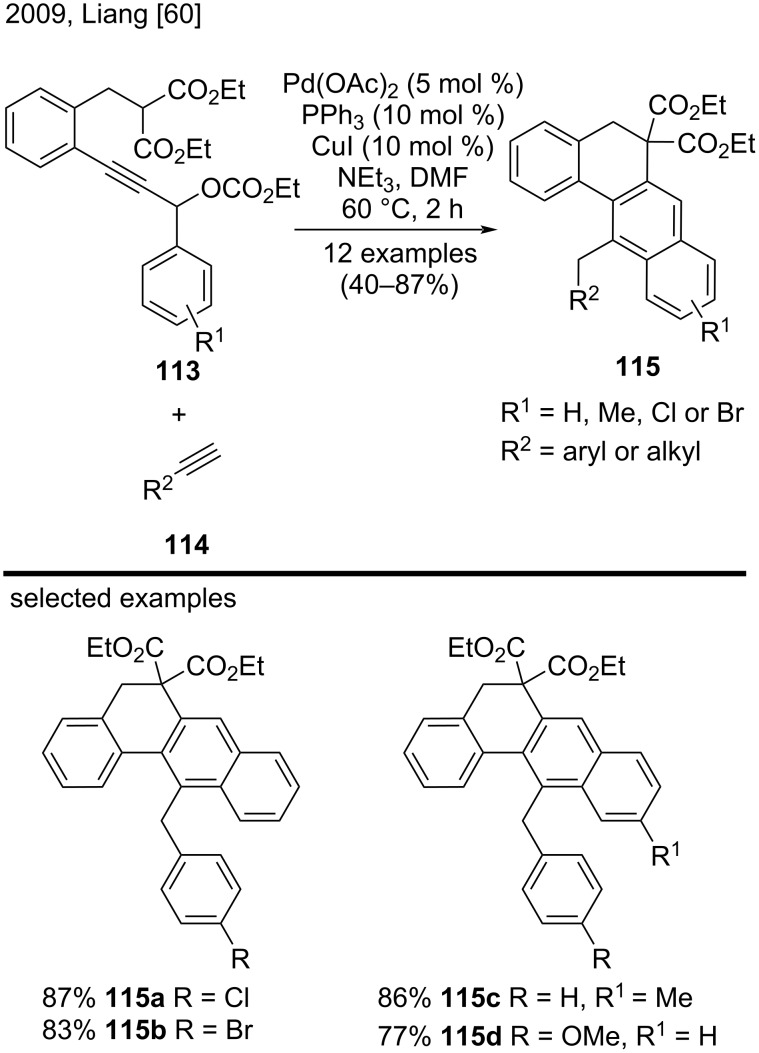
Palladium-catalyzed tandem C–H activation/bis-cyclization of propargylic carbonates.

In 2009, Liang et al. reported an efficient and highly regioselective route to construct substituted tetracyclic benz[*a*]anthracene derivatives **115** (Scheme 26) [60]. For this purpose, the authors developed an efficient palladium-catalyzed tandem C–H activation/bis-cyclization reaction of propargylic carbonates **113** with terminal alkynes **114**. The scope of this reaction consisted of 12 examples that were synthesized in moderate to good yields (40–87%). The authors obtained the best yields by using electron-deficient aryl alkynes or secondary carbonates with electron-rich arene substituents (**115a**–**d**). The authors also employed aliphatic alkynes in this methodology, but they obtained lower yields [60].

**Scheme 27:**
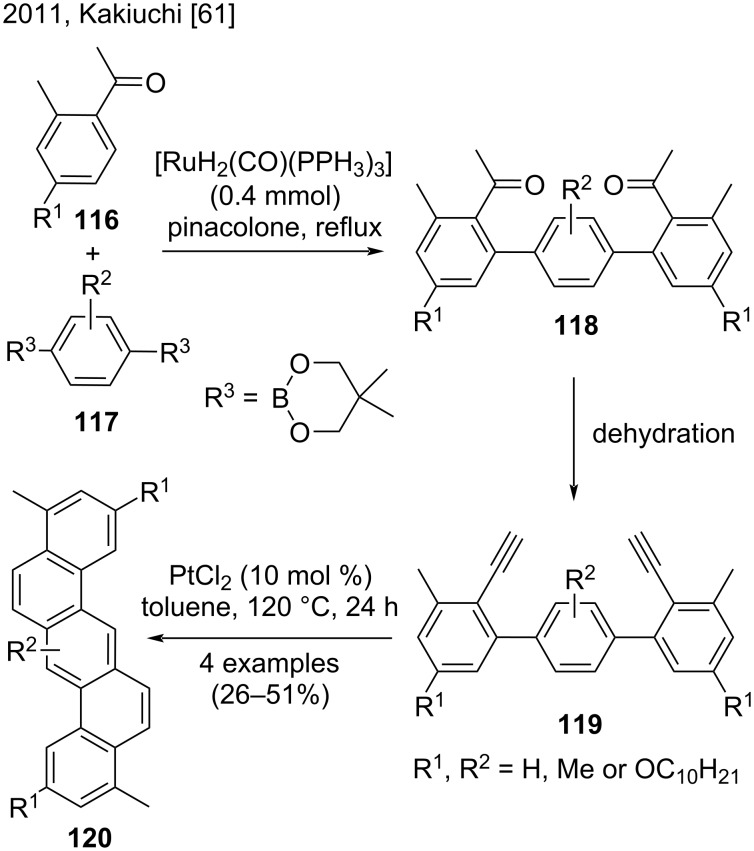
Ruthenium-catalyzed C–H arylation of acetophenone derivatives with arenediboronates.

In a study published in 2011, Kakiuchi and co-workers reported a new method to synthesize dibenzo[*a*,*h*]anthracenes and picene derivatives by a ruthenium-catalyzed regioselective C–H arylation of aromatic ketones (Scheme 27) [61]. The authors coupled acetophenone derivatives **116** and 1,4-benzenediboronates **117** at a 2:1 ratio, to obtain *p*-terphenyl derivatives **118**. In the second step, the conversion of the acetyl group of compounds **118** to an ethynyl group afforded diethynylterphenyls **119**. In the last step, a platinum-catalyzed intramolecular cycloaromatization of **119** provided dibenzo[*a*,*h*]anthracenes **120** bearing methyl and alkoxy groups in low to moderate yields (26–51%) [61].

#### Metal-catalyzed intramolecular double-cyclization

**Scheme 28:**
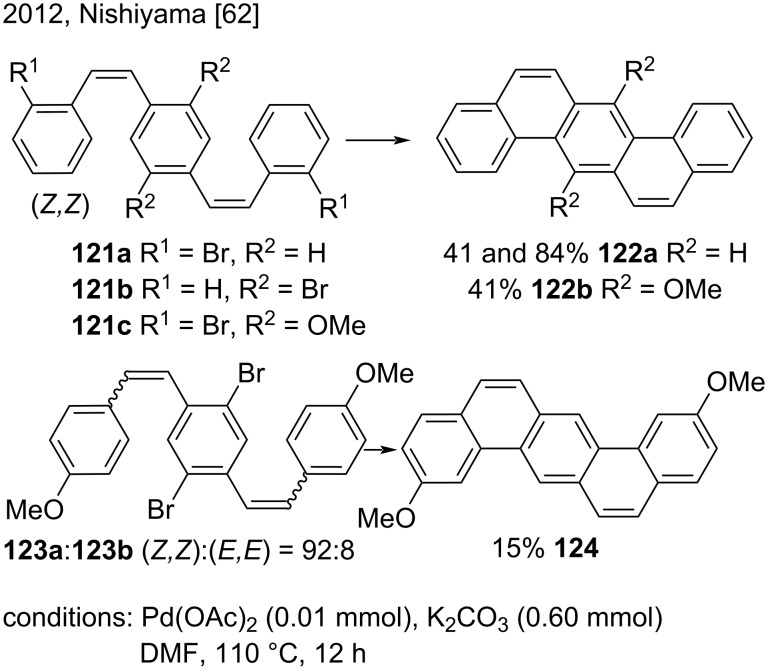
Pd-catalyzed intramolecular cyclization of (*Z,Z*)-*p*-styrylstilbene derivatives.

In 2012, Nishiyama’s research group synthesized dibenzo[*a*,*h*]anthracenes by the Pd-catalyzed intramolecular double-cyclization of *p*-styrylstilbene derivatives for the first time (Scheme 28) [62]. The authors prepared dibenzo[*a,h*]anthracene (**122a** (41 and 84% yield) and two derivatives **122b** (41% yield), and **124** (15% yield) via Pd-catalyzed intramolecular double-cyclization of the corresponding (*Z,Z*)-*p*-styrylstilbene derivatives **121a**–**c** and **123a**, prepared according to the literature. However, when they carried out the reaction by using (*E,E*)-**121a** instead of (*Z,Z*)-**121a** under the same conditions, cyclization did not occur, so they did not detect the corresponding dibenzo[*a,h*]anthracene [62].

**Scheme 29:**
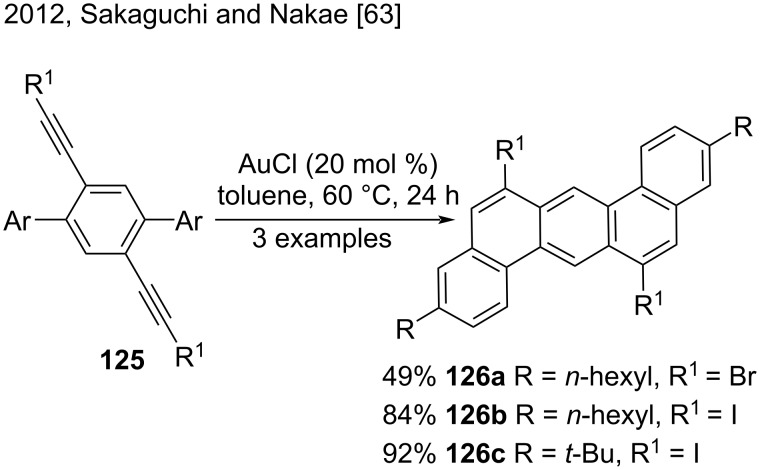
AuCl-catalyzed double cyclization of diiodoethynylterphenyl compounds.

Sakaguchi, Nakae, and co-workers synthesized halogenated dibenzo[*a,h*]anthracenes **126a**–**c** and 5,8-diiodopicenes from dibromo- or diiodoethynylterphenyl compounds **125** via a AuCl-catalyzed intramolecular double cyclization (Scheme 29) [63]. The authors investigated the regioselectivity of the double cyclization and concluded that the reaction strongly depended on the position of the ethynyl groups in the terphenyl compounds. Terphenyls **125** were the most appropriate to prepare dibenzo[*a,h*]anthracenes in good yield (49–92%). AuCl was a notable catalyst because it maintained high cyclization activity for iodoethynyl groups [63].

#### Intramolecular cyclization

**Scheme 30:**
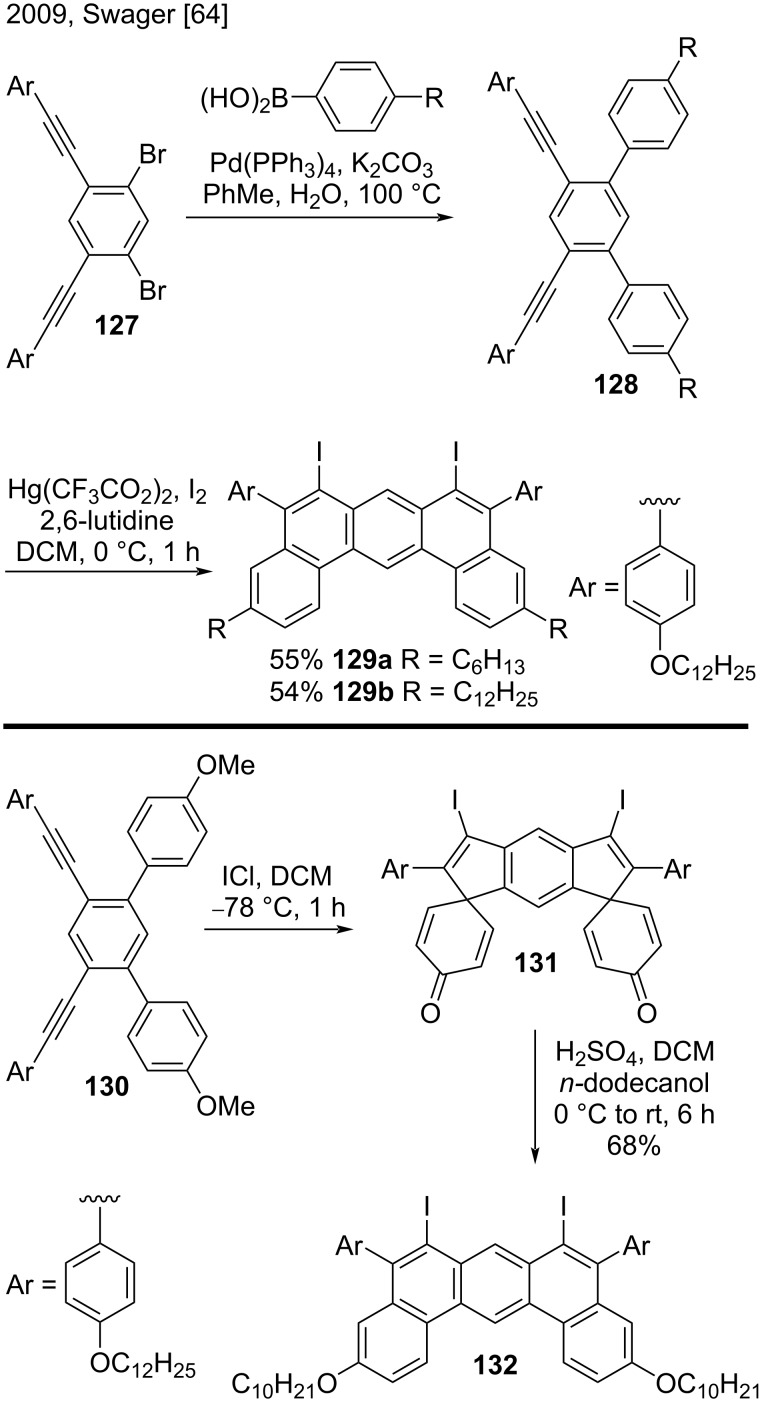
Iodonium-induced electrophilic cyclization of terphenyl derivatives.

In 2009, Swager’s research group published the synthesis of fluorescent macrocycles based on 1,3-butadiyne-bridged dibenzo[*a*,*j*]anthracene subunits via a multistep route (Scheme 30) [64]. They synthesized substituted dibenzo[*a*,*j*]anthracenes **129a** and **129b** in two steps. First, they subjected dibromides **127** to a double Suzuki coupling with 4-alkylphenylboronic acids, to obtain the terphenyl derivatives **128**. Then, the authors converted compounds **128**, in moderate yield (54–55%), to the corresponding 6,8-diiododibenzo[*a*,*j*]anthracenes **129a** and **129b** via double iodonium-induced electrophilic cyclization. The terphenyl **130** was converted to the diiododibenzo[*a*,*j*]anthracene derivative **132** in two steps comprising cyclization and further treatment of intermediate **131** with sulfuric acid [64].

**Scheme 31:**
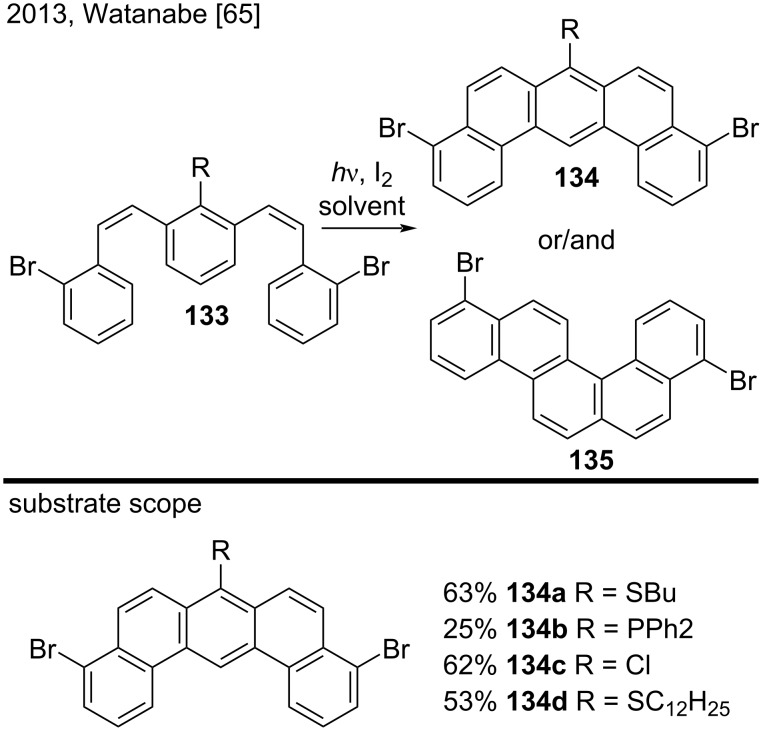
Oxidative photocyclization of 1,3-distyrylbenzene derivatives.

Watanabe and co-workers investigated the oxidative photocyclization of 1,3-distyrylbenzene derivatives **133** bearing different substituents at position 2 in order to find efficient blocking groups to afford dibenzoanthracene derivatives (Scheme 31) [65]. For the first time, their results established that butylthio and diphenylphosphino groups performed well as blocking groups, to give the corresponding substituted dibenzo[*a*,*j*]anthracenes **134a** and **134b**, respectively. The authors also used 1,3-distyrylbenzene **133** substituted with chloro and dodecylthio groups and obtained the dibenzo[*a*,*j*]anthracenes **134c** and **134d** in moderate yields (53–62%). However, when they employed 1,3-distyrylbenzenes substituted with methyl, trimethylsilyl, dimethylamino, butoxy, or fluoro groups, they only obtained compounds similar to benzo[*c*]chrysene derivative **135** [65].

**Scheme 32:**
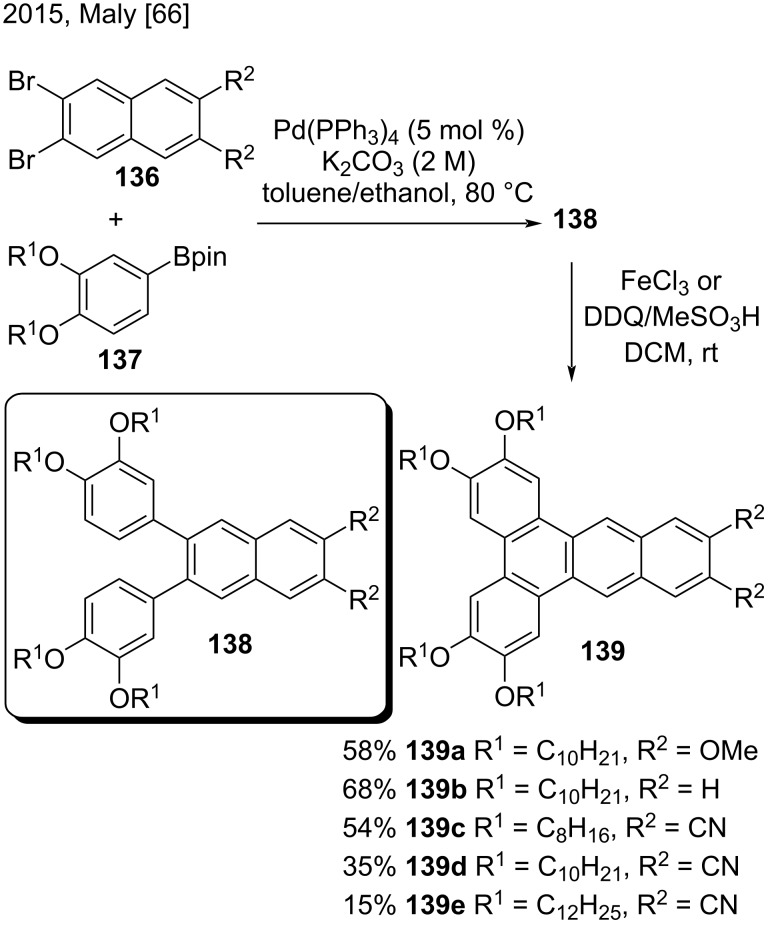
Oxidative cyclization of 2,3-diphenylnaphthalenes.

In 2015, Maly and co-workers reported a two-step synthetic route to obtain substituted 2,3,5,6-tetraalkoxydibenzo[*a*,*c*]anthracenes **139** bearing H, CN, or OMe groups at positions 11 and 12, to study their liquid crystalline properties (Scheme 32) [66]. Their methodology started with a Suzuki coupling reaction of substituted dibromonaphthalenes **136** and boronate diesters **137**, to provide the corresponding 2,3-diphenylnaphthalenes **138**. Then, an oxidative cyclization of **138** in the presence of FeCl_3_ afforded dibenzo[*a*,*c*]anthracenes **139a** and **139b** in moderate yields (58–68%). On the other hand, they obtained dibenzo[*a*,*c*]anthracenes **139c**–**e** in low to moderate yields (15–54%) when they used DDQ/MeSO_3_H instead of FeCl_3_ [66].

**Scheme 33:**
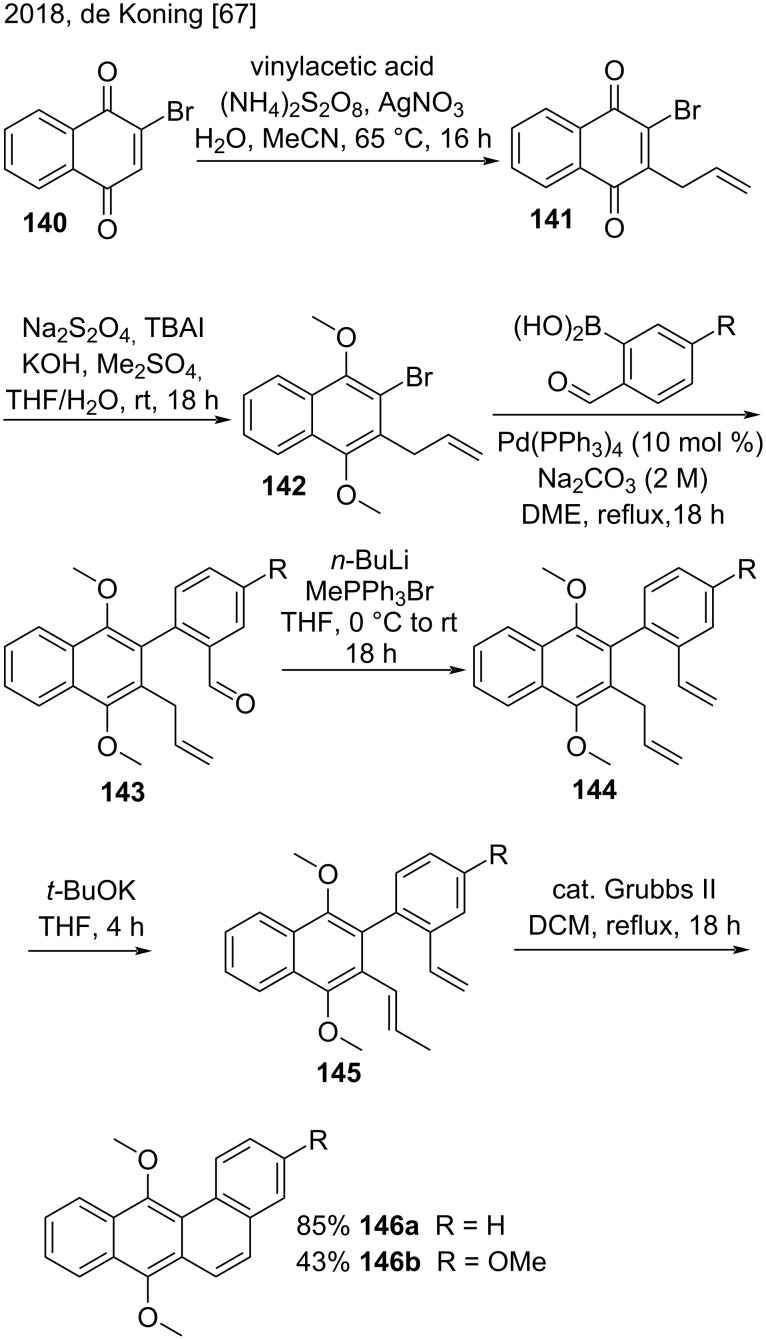
Suzuki-Miyaura/isomerization/ring closing metathesis strategy to synthesize benz[*a*]anthracenes.

In a study published in 2018, de Koning and co-workers reported a new methodology to synthesize the benzo[*a*]anthracene skeleton of angucycline derivatives **146** by using a multistep strategy based on Suzuki–Miyaura, isomerization, and ring-closing metathesis reactions (Scheme 33) [67]. The commercially available 2-bromonaphthoquinone (**140**) reacted with vinylacetic acid to afford the allylated compound **141**. Then, reduction of **141** and sequential *O*-methyl-protection furnished naphthalene **142**. A Suzuki–Miyaura coupling reaction between **142** and boronic acids afforded 2-naphthylbenzaldehydes **143**, which were further subjected to a Wittig reaction affording naphthalenes **144**. Isomerization of compounds **144** produced substituted styrenes **145**. With the styrenes **145** in hands, the authors employed the Grubbs II catalyst-promoted ring closure to obtain the benzo[*a*]anthracenes **146a** (85% yield) and **146b** (43% yield) [67].

#### Other procedures

**Scheme 34:**
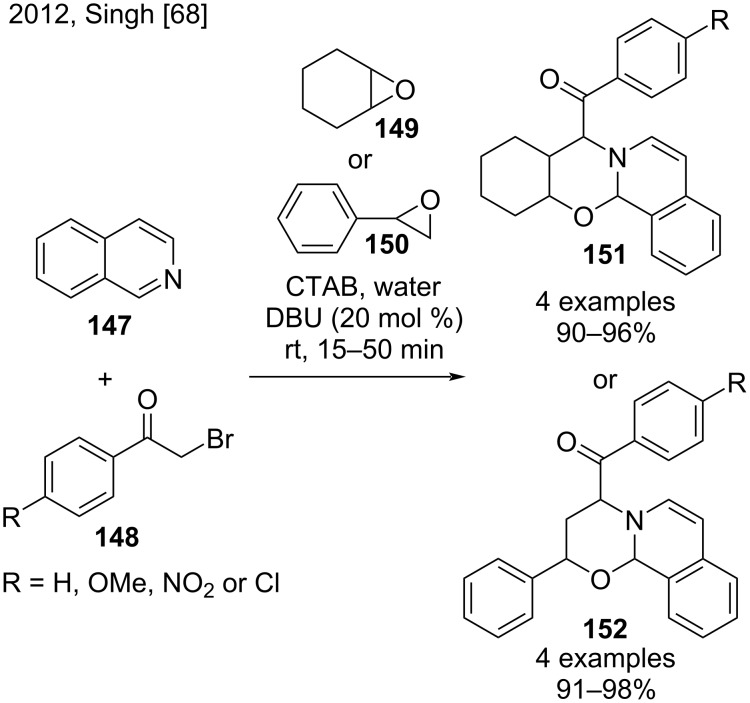
Green synthesis of oxa-aza-benzo[*a*]anthracene and oxa-aza-phenanthrene derivatives.

In 2012, Singh and co-workers performed green syntheses of oxa-aza-benzo[*a*]anthracene and oxa-aza-phenanthrene derivatives **151** and **152** via a sequential one-pot reaction in an aqueous micellar system (Scheme 34) [68]. This methodology comprised reactions of isoquinoline (**147**), phenacyl bromides **148** bearing OMe, NO_2_, or Cl groups, and epoxides (**149** and **150**), DBU as catalyst, CTAB as surfactant, and water as solvent. The use of cyclohexene oxide (**149**) provided oxa-aza-benzo[*a*]anthracene derivatives **151** in excellent yields (90–96%). On the other hand, the use of styrene oxide (**150**) provided oxa-aza-phenanthrene derivatives **152**, also in excellent yields (91–98%) [68].

**Scheme 35:**
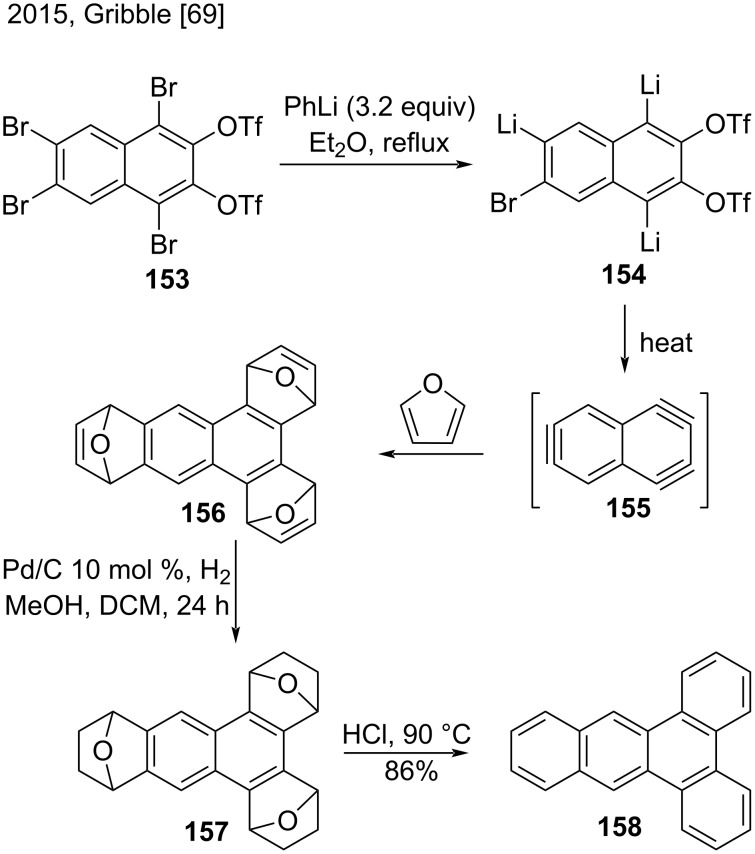
Triple benzannulation of substituted naphtalene via a 1,3,6-naphthotriyne synthetic equivalent.

In 2015, Gribble et al. published a new method to synthesize dibenzo[*a*,*c*]anthracene (**158**) based on a triple benzannulation of naphthalene derivative **153** via a 1,3,6-naphthotriyne synthetic equivalent **155** (Scheme 35) [69]. First, reaction of brominated naphthalene **153** with PhLi yielded compound **154**, which collapsed to naphthotriyne **155** at elevated temperatures. Sequential addition of furan generated the trisadduct **156**. Then, dibenz[*a,c*]anthracene **158** was obtained in good yield (86%) in two steps by hydrogenation of **156** and further dehydration of **157** under reflux with concentrated HCl [69].

### Synthesis of anthraquinone derivatives

#### Friedel–Crafts intramolecular cyclization

**Scheme 36:**
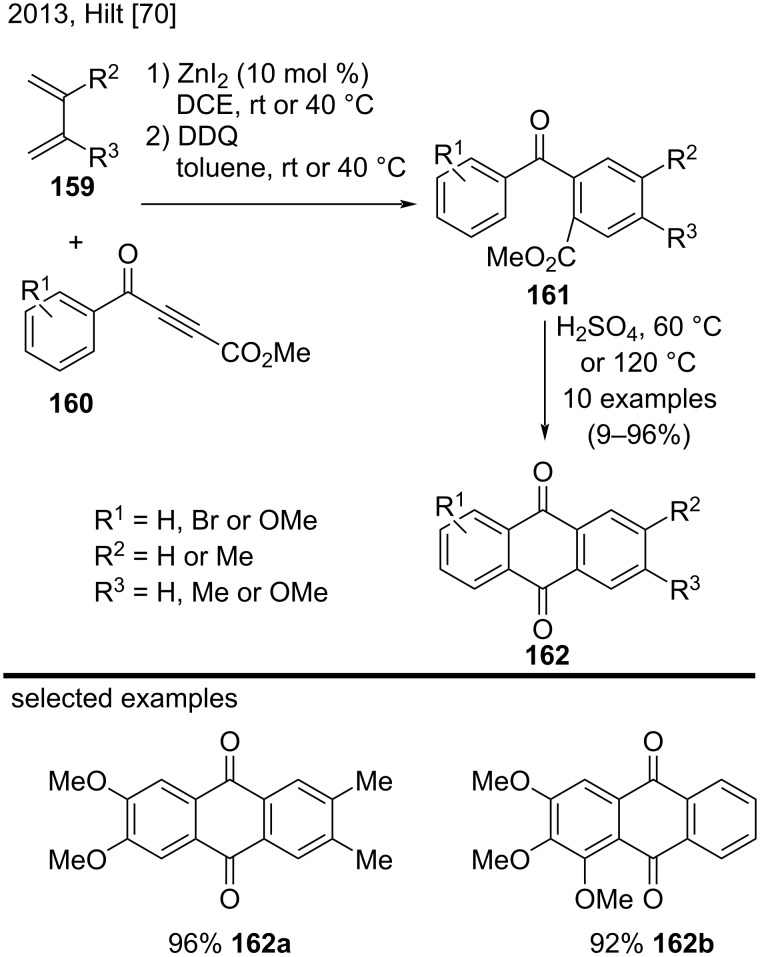
Zinc iodide-catalyzed Diels–Alder reactions with 1,3-dienes and aroyl propiolates followed by intramolecular Friedel-Crafts cyclization.

In 2013, Hilt and co-workers synthesized symmetric and asymmetric anthraquinone derivatives **162** bearing Br, Me, or OMe groups via ZnI_2_-catalyzed Diels–Alder reactions/DDQ oxidation of 1,3-dienes **159** and aroyl-substituted propiolates **160** (Scheme 36) [70]. Subsequently, the authors performed an intramolecular Friedel-Crafts cyclization of the corresponding derivatives **161** by using concentrated sulfuric acid. The authors noted that the more electron-donating alkyl or methoxy groups were present in the aromatic ring, the more efficient the Friedel–Crafts-type cyclization would be. Representative examples included substituted anthraquinones **162a** and **162b,** that were obtained in very good yields (92–96%) [70].

**Scheme 37:**
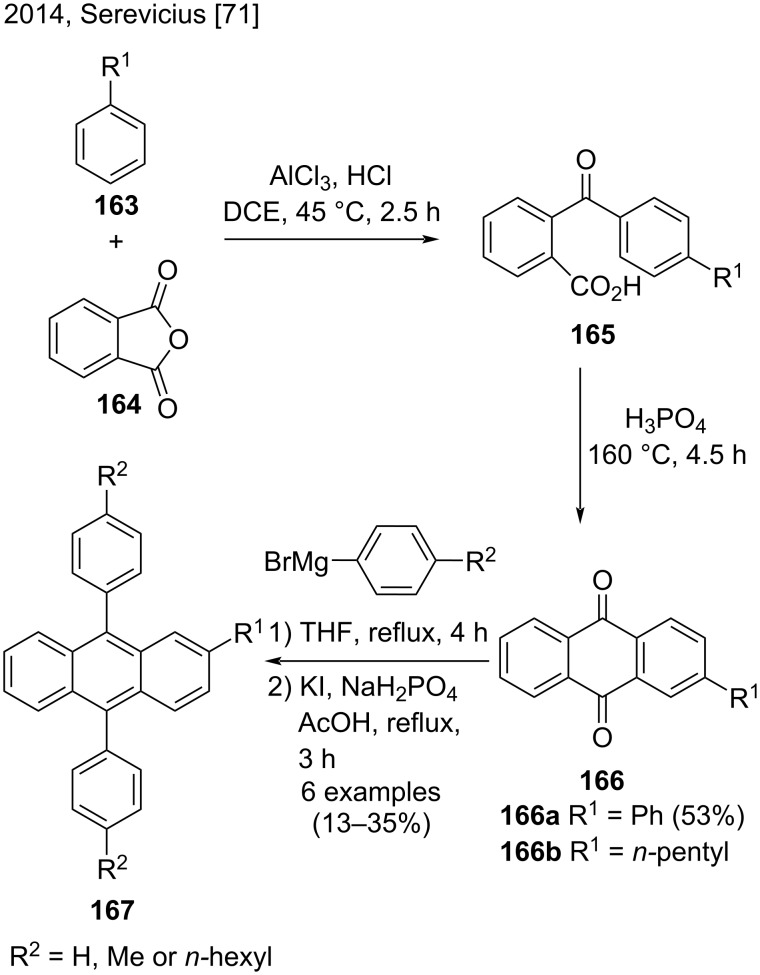
H_3_PO_4_-promoted intramolecular cyclization of substituted benzoic acids.

In 2014, Serevicius and co-workers synthesized two derivatives of substituted anthraquinones **166** aiming at further preparation of 9,10-diphenyltanthracenes **167** (Scheme 37) [71]. First, they reacted arenes **163** with phthalic anhydride (**164**) in the presence of aluminum chloride and hydrochloric acid, to obtain benzoylbenzoic acid derivatives **165**. Then, the H_3_PO_4_-promoted intramolecular cyclization of **165** led to anthraquinones **166a** and **166b**, which reacted with arylmagnesium bromides to afford the substituted 9,10-diphenylanthracenes **167** in low yields (13–35%). Despite that, this strategy was particularly interesting for the synthesis of anthraquinone derivatives by using different arenes [71].

**Scheme 38:**
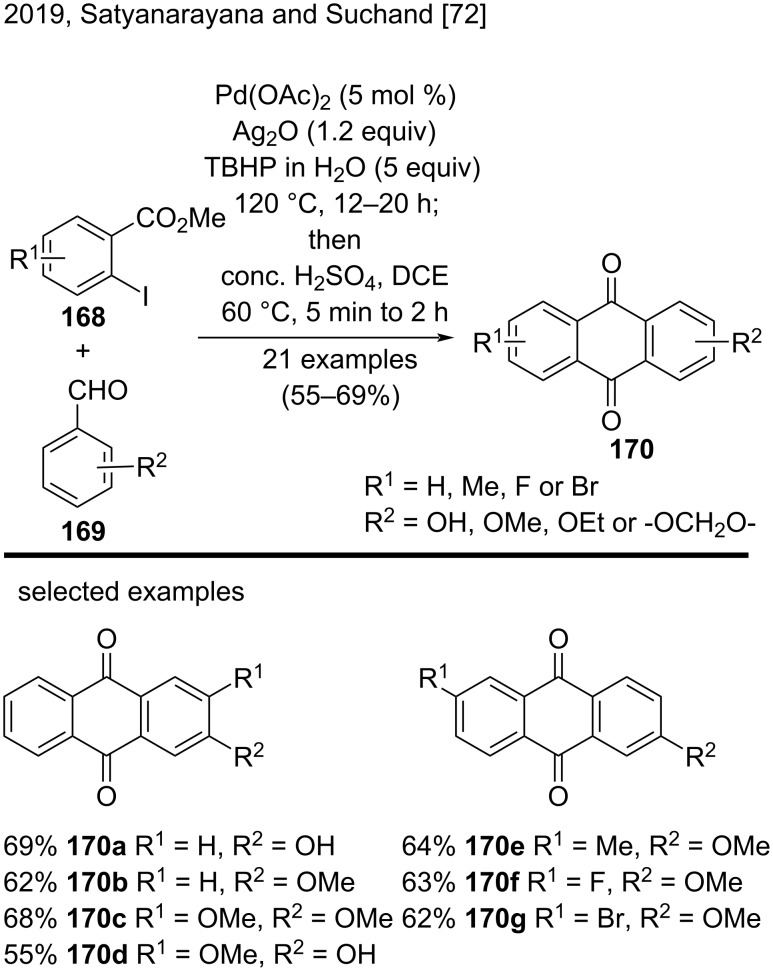
Palladium-catalyzed intermolecular direct acylation of aromatic aldehydes and *o-*iodoesters.

Recently, Satyanarayana and Suchand developed a one-pot strategy to synthesize substituted anthraquinones **170** via palladium-catalyzed intermolecular direct acylation of aromatic aldehydes **169** and *o-*iodoesters **168** (Scheme 38) [72]. The overall process involved sequential Pd-catalyzed intermolecular acylation/H_2_SO_4_-promoted intramolecular Friedel–Crafts cyclization. The authors prepared substituted anthraquinones bearing Me, OMe, OH, Br, F, or other groups, such as anthraquinones **170a**–**g**, in moderate to good yields (55–69%). The reaction proved efficient mainly with electron-donating substituents on the benzaldeydes **169** [72].

#### Cycloaddition reactions

**Scheme 39:**
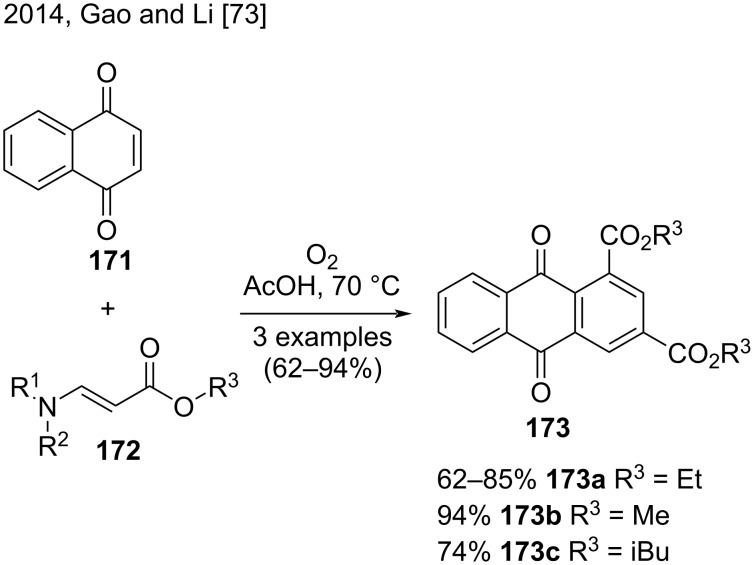
Cycloaddition/oxidative aromatization of quinone and β-enamino esters.

In 2014, Gao, Li, and their co-workers published a facile strategy to synthesize polysubstituted aromatic compounds from the reaction of quinones or maleimides with β-enamino esters (Scheme 39) [73]. They synthesized anthraquinone derivatives **173** in good yield (62–94%) via a cycloaddition/oxidative aromatization sequence involving quinone **171** and substituted β-enamino esters **172** as precursors. They prepared anthraquinone **173a** starting from three different β-enamino esters; a less bulky β-enamino ester favored the reaction. The scope of the reaction also included anthraquinones **173b** and **173c**, obtained in good yields (74–94%) [73].

**Scheme 40:**
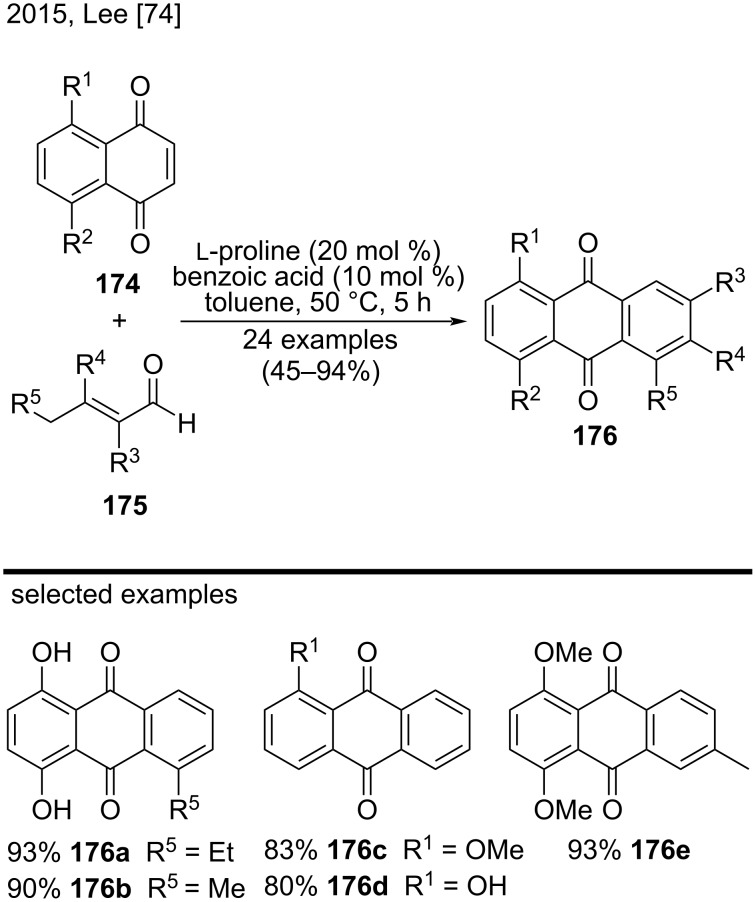
ʟ-Proline-catalyzed [4 + 2] cycloaddition reaction of naphthoquinones and α,β-unsaturated aldehydes.

In 2015, in a related approach, Lee et al. disclosed a direct one-pot synthesis of anthraquinones and tetracenediones by using ʟ-proline as organocatalyst and benzoic acid as additive (Scheme 40) [74]. They synthesized substituted anthraquinones bearing Me, Et, or hydroxy groups, such as compounds **176a**–**e**, in moderate to good yields (45–94%) through a [4 + 2] cycloaddition reaction of 1,4-substituted naphthoquinones **174** and α,β-unsaturated aldehydes **175** catalyzed by ʟ-proline. During optimization studies, the authors observed that benzoic acid played an important role in the formation of quinone compounds, increasing the yields and decreasing reaction times [74].

**Scheme 41:**
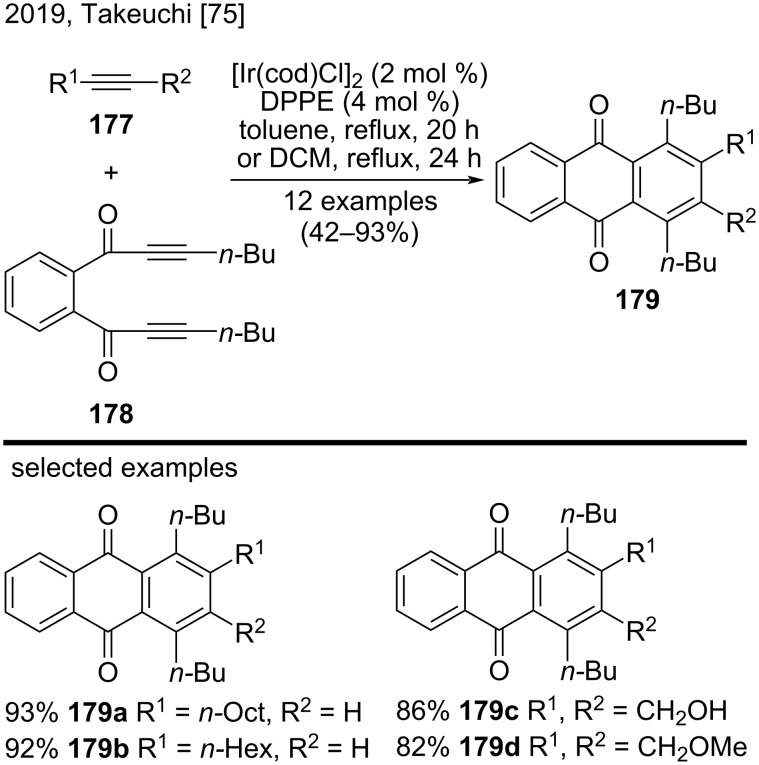
Iridium-catalyzed [2 + 2 + 2] cycloaddition of a 1,2-bis(propiolyl)benzene derivative with alkynes.

Recently, Takeuchi’s research group synthesized polysubstituted anthraquinones **179** in moderate to good yields (42–93%) via an iridium-catalyzed [2 + 2 + 2] cycloaddition of 1,2-bis(propiolyl)benzene derivative **178** and terminal/internal alkynes **177** (Scheme 41) [75]. The authors performed the reactions with terminal alkynes in toluene, and reactions with internal alkynes in dichloromethane (DCM). They noted that the use of 1,2-bis(diphenylphosphino)ethane (DPPE) as ligand improved the yield of the anthraquinones. Representative examples included anthraquinones **179a** and **179b** obtained from terminal alkynes and **179c** and **179d** from internal alkynes [75].

#### Multicomponent reactions

**Scheme 42:**
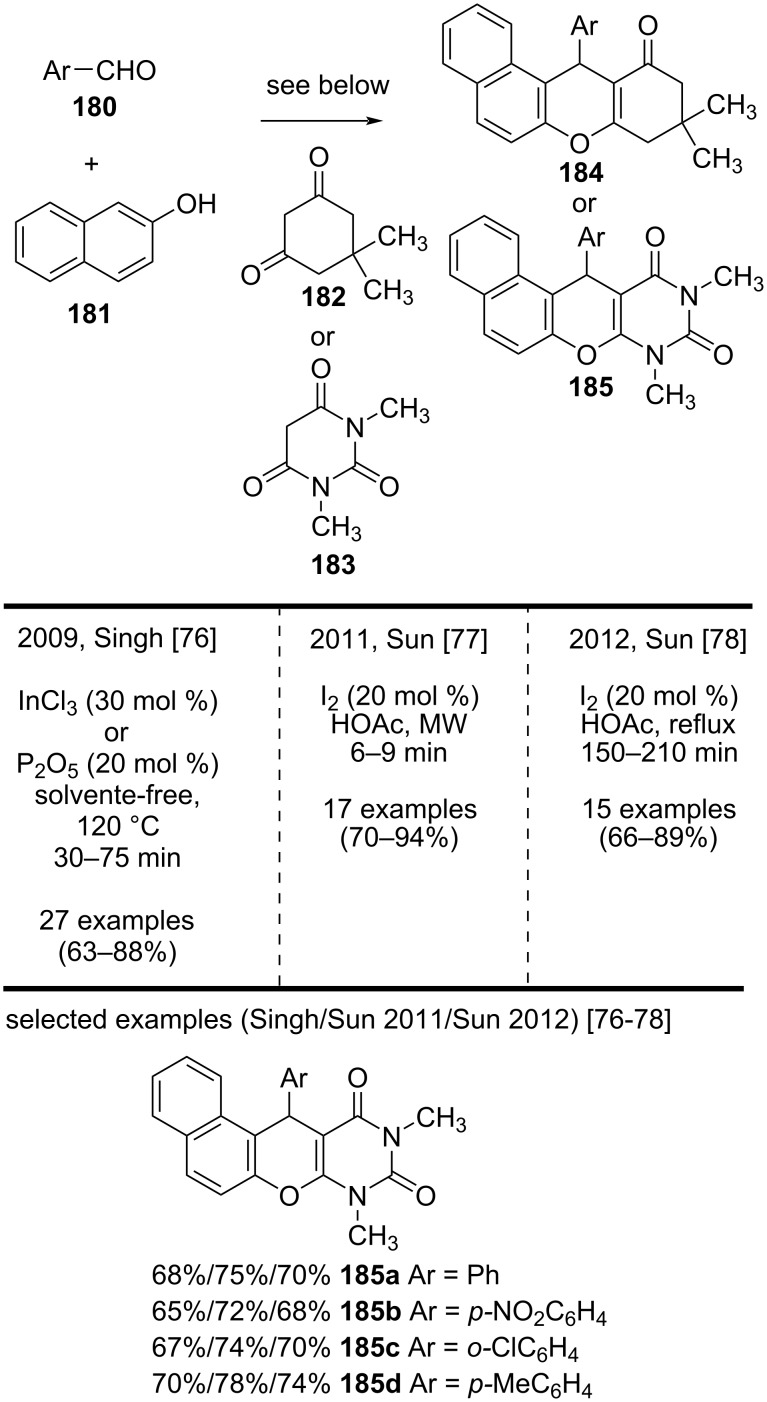
Synthesis of several anthraquinone derivatives by using InCl_3_ and molecular iodine.

In 2009, Singh and co-workers reported a solvent-free methodology to synthesize tetrahydrobenzo[*a*]xanthene-11-ones **184** and diazabenzo[*a*]anthracene-9,11-dione derivatives **185** in good yields via a multicomponent reaction (Scheme 42) [76]. This methodology was based on the cyclocondensation of aromatic aldehydes **180**, β-naphthol (**181**), and cyclic 1,3-dicarbonyl compounds **182** or **183**, catalyzed by InCl_3_ or P_2_O_5_. The authors achieved the best results (63–88% yield) when they carried out the reactions with InCl_3_ instead of P_2_O_5._ However, the reactions with some aliphatic aldehydes such as cinnamaldehyde, isobutyraldehyde, and cyclohexanecarboxaldehyde did not generate the expected products [76]. Sun and co-workers modified the method proposed by Singh. In 2011, they reported a method that employed molecular iodine as the catalyst, under microwave radiation as heat source, and obtained tetrahydrobenzo[*a*]xanthene-11-one and diazabenzo[*a*]anthracene-9,11-dione derivatives in good to excellent yields (70–94%) [77]. Then, in 2012, Sun and co-workers reported another method employing molecular iodine as catalyst under reflux with acetic acid instead of microwave radiation and also obtained good yields (66–89%) [78]. Because the three methodologies provided good yields, the authors were able to synthesize substituted derivatives, such as compounds **184** and **185**, bearing diverse aryl groups, derived from aromatic aldehydes. However, the direct comparison among the yields of diazabenzo[*a*]anthracene-9,11-diones **185a**–**d** shows that the methodology developed in 2011 by Sun and co-workers [77] provided better results.

**Scheme 43:**
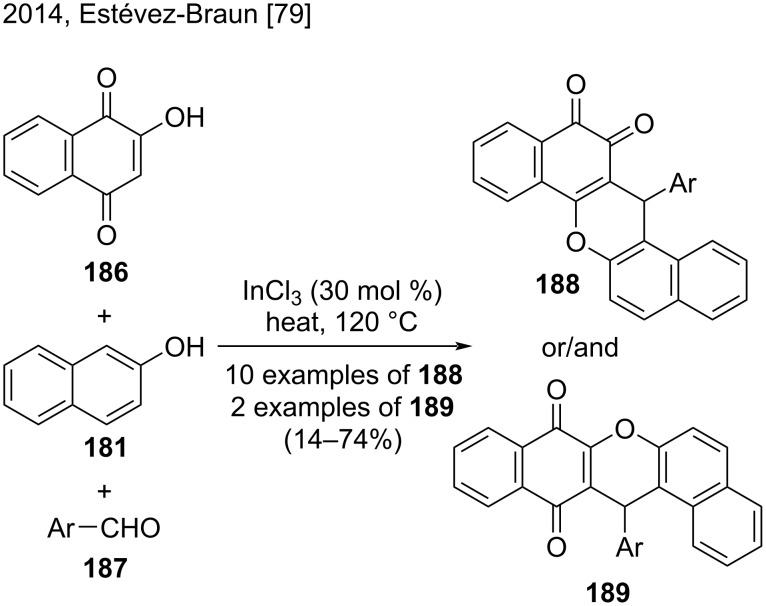
Indium-catalyzed multicomponent reactions employing 2-hydroxy-1,4-naphthoquinone (**186**), β-naphthol (**181**), and aromatic aldehydes.

In a related approach, Estévez-Braun and co-workers synthesized dibenzo[*a,h*]anthracene-12,13-diones **188** from 2-hydroxy-1,4-naphthoquinone (**186**), β-naphthol (**181**), and aromatic aldehydes **187** through a multicomponent reaction that used InCl_3_ as catalyst under solvent-free conditions (Scheme 43) [79]. The authors used various heteroaromatic aldehydes and substituted aromatic aldehydes containing electron-donating and electron-withdrawing substituents, to obtain the *ortho* adducts **188** in variable yields (14–74%). The use of pyridine-3-carbaldehyde and 2,4,5-trimethoxybenzaldehyde also afforded the corresponding *para*-adducts **189** in different proportions in the reaction mixture. As expected, when the authors used aliphatic aldehydes, they did not detect the corresponding derivatives **188** [79].

#### Other procedures

**Scheme 44:**
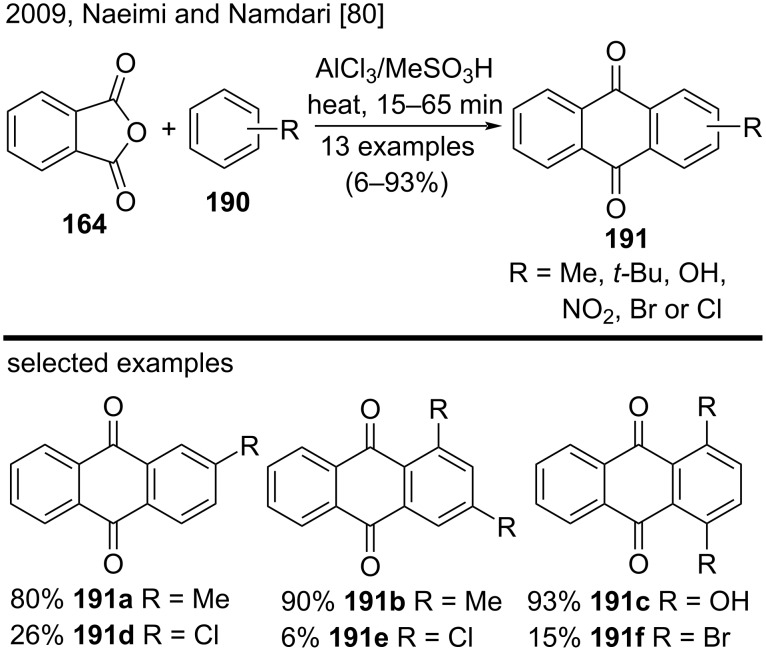
Synthesis of substituted anthraquinones catalyzed by an AlCl_3_/MeSO_3_H system.

In 2009, Naeimi and Namdari published a one-pot synthesis of substituted anthraquinone derivatives **191** from phthalic anhydride (**164**) and several arenes **190** by using a combined system of AlCl_3_ and MeSO_3_H (Scheme 44) [80]. Arenes **190** containing electron-donating groups yielded the anthraquinone derivatives **191a**–**c** in very good yields (80–93%). On the other hand, reactions involving arenes **190** bearing electron-withdrawing substituents afforded the anthraquinone derivatives **191d**–**f** in significantly lower yields (6–26%) [80].

**Scheme 45:**
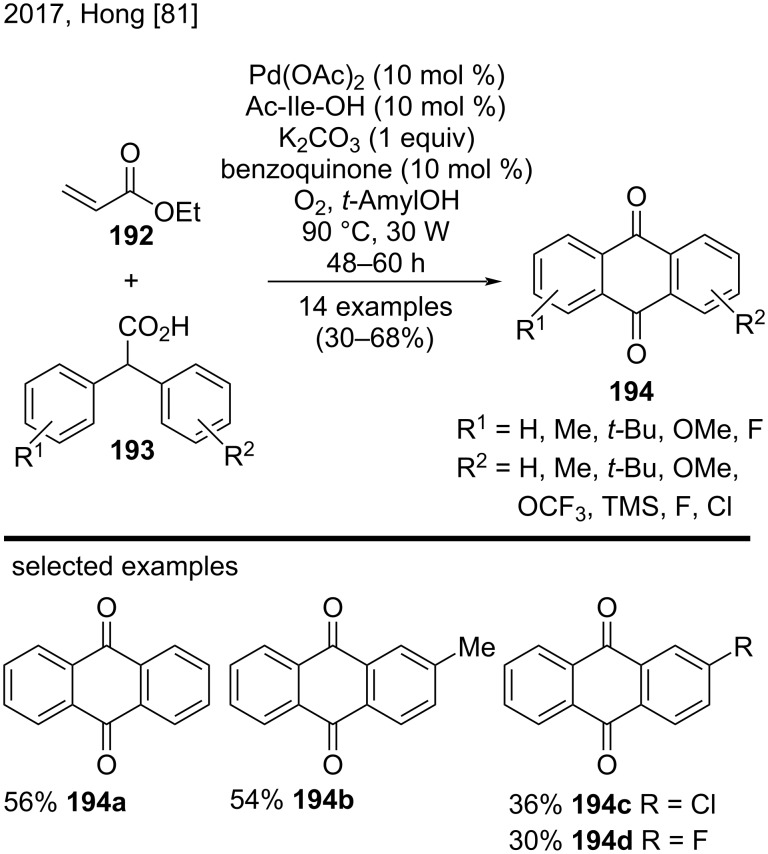
Palladium(II)-catalyzed/visible light-mediated synthesis of anthraquinones.

In 2017, Hong and co-workers reported another efficient protocol for the direct synthesis of substituted anthraquinones (Scheme 45) [81]. The authors employed palladium(II) acetate and visible light under O_2_ in the reactions between ethyl acrylate (**192**) and substituted diaryl carboxylic acids **193**, to produce anthraquinones **194** in low to moderate yields (30–68%). In a direct comparison with the methodology proposed by Hong for the synthesis of anthracenes, previously shown in Scheme 7 [41], the key to obtaining anthraquinones was the photooxidation induced by visible light, which afforded the substituted anthraquinones **194**. In this case, the effect of the aromatic ring substituents also affected the yield of the anthraquinones, as can be seen from the representative examples **194a**–**d** [81].

**Scheme 46:**
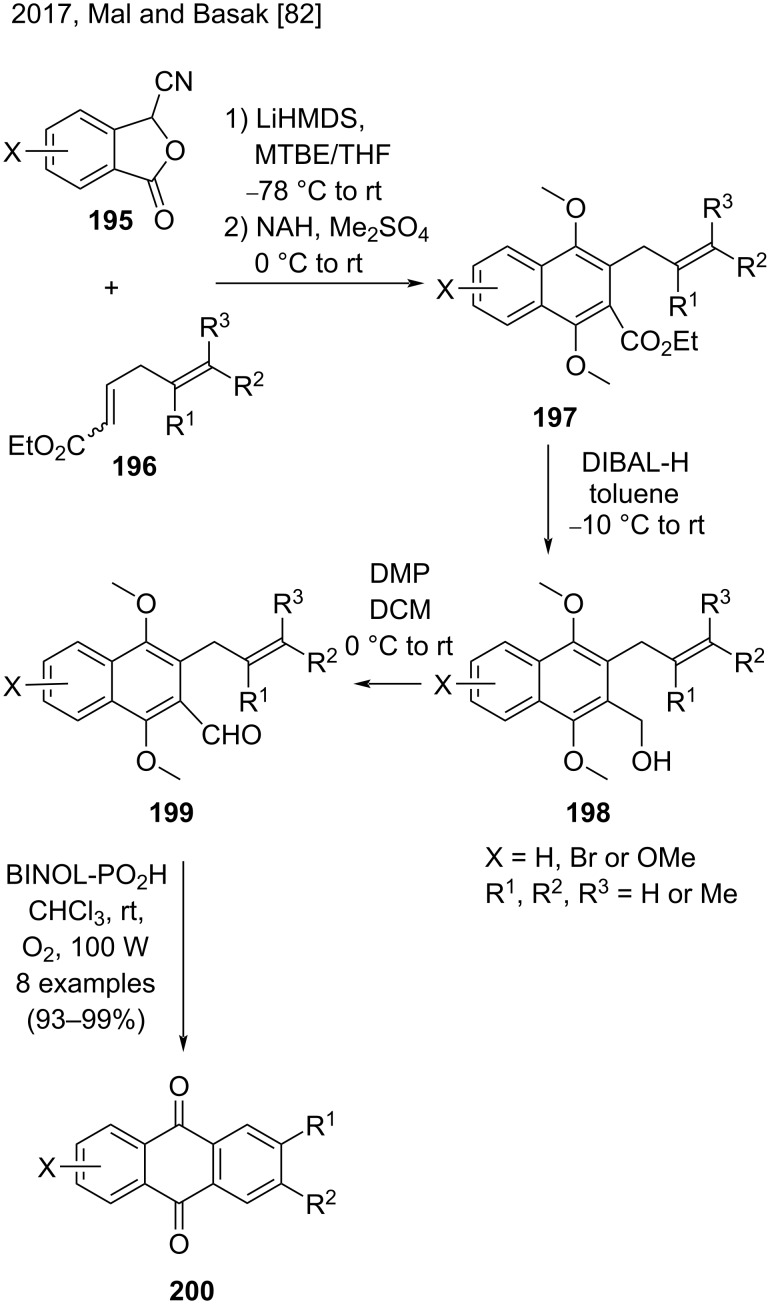
[4 + 2] Anionic annulation reaction for the synthesis of substituted anthraquinones.

In 2017, Mal and Basak applied a [4 + 2] anionic annulation of substituted cyanophthalides **195** with dienoates **196** and obtained 3-allylnaphthoates **197** (Scheme 46) [82]. Then, they converted the naphthoate derivatives **197** to the corresponding alcohols **198** by DIBAL-H reduction, and later to aldehydes **199** by Dess–Martin periodinane (DMP) oxidation. The aldehydes **199** were treated with BINOL-PO_2_H in chloroform, in the presence of air and light, to produce the corresponding anthraquinones **200** in excellent yields (93–99%) [82].

In this review, a more detailed discussion of different types of anthracene derivatives (e.g., anthracenophanes) that have been the subject of research interest for some time [1] cannot be included. Nevertheless, readers interested in this area should refer to several interesting articles on this subject such as the work of the research groups of Bettinger [83–84], Ohmori [85], and Novak [86].

## Conclusion

In this review, we have highlighted the most recent preparative methods for anthracene derivatives. Among the many synthetic strategies reported in the last twelve years, metal-catalyzed/promoted reactions, especially those with internal alkynes, have been the most used, possibly because they provide a simple and straightforward ring extension method to construct polycyclic aromatic compounds, especially anthracenes with substituents in the 2-, 3-, 6-, and 7-positions. Also, considering the difficulties and limitations of direct syntheses of anthracene derivatives, the considerable number of methodologies reported in recent years is truly surprising. Due to the wide applicability of anthracene and anthraquinone derivatives in important fields of science, the development of new synthetic methods is likely to increase. We hope that this review can serve to guide and to inspire future advances in synthetic organic chemistry for this kind of polycyclic compounds.
